# Asymmetric synthesis of CF_2_-functionalized aziridines by combined strong Brønsted acid catalysis

**DOI:** 10.3762/bjoc.16.60

**Published:** 2020-04-07

**Authors:** Xing-Fa Tan, Fa-Guang Zhang, Jun-An Ma

**Affiliations:** 1 Department of Chemistry, Tianjin Key Laboratory of Molecular Optoelectronic Sciences, and Tianjin Collaborative Innovation Centre of Chemical Science & Engineering, Tianjin University, Tianjin 300072, China; 2 Joint School of NUS & TJU, International Campus of Tianjin University, Fuzhou 350207, China

**Keywords:** aziridines, chiral disulfonimides, difluoromethyl compounds, fluorinated diazo reagents, strong Brønsted acids

## Abstract

A diastereo- and enantioselective approach to access chiral CF_2_-functionalized aziridines from difluorodiazoethyl phenyl sulfone (PhSO_2_CF_2_CHN_2_) and in situ-formed aldimines is described. This multicomponent reaction is enabled by a combined strong Brønsted acid catalytic platform consisting of a chiral disulfonimide and 2-carboxyphenylboronic acid. The optical purity of the obtained CF_2_-substituted aziridines could be further improved by a practical dissolution–filtration procedure.

## Introduction

**Scheme 1:**
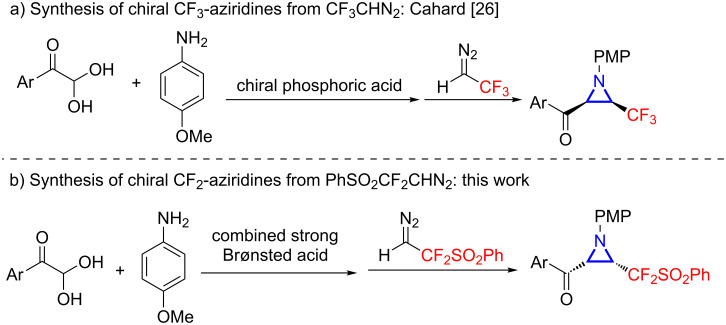
Preparation of chiral aziridines from fluorinated diazo reagents.

Chiral aziridines are prevalently found in natural products and artificially made bioactive molecules, thus receiving significant attention in the past decades [1–6]. Among them, the introduction of fluorine or fluoroalkyl groups into three-membered N-heterocycles has emerged as an attractive direction due to the unique fluorine effect in pharmaceuticals and biology [7–11]. In this context, it is not surprising that the syntheses of trifluoromethylaziridines have been pursued from versatile precursors [12–25]. However, catalytic asymmetric approaches to chiral CF_3_-functionalized aziridines have only been reported by Cahard in 2012, who utilized trifluorodiazoethane (CF_3_CHN_2_) as the nucleophile to react with aldimines catalyzed by chiral phosphoric acid (Scheme 1a) [26]. In comparison, there is a significant dearth of available synthetic approaches to CF_2_-functionalized aziridines, particularly in a stereocontrolled manner. Indeed, a handful of reported methods document the employment of difluoromethylimines, difluoromethyl phenyl sulfone, and difluoromethyl vinyl sulfonium salts as the fluorinating partner en route to various CF_2_-substituted aziridines [27–31], and a general protocol to chiral CF_2_-aziridines remains an unsolved challenge. Thus, herein we report a diastereo- and enantioselective aza-Darzens reaction between in situ-generated aldimines and our recently developed difluorodiazo reagent PhSO_2_CF_2_CHN_2_ acting as the difluorinated nucleophile [32–35], providing access to a variety of chiral CF_2_-fuctionalized aziridines under mild conditions (Scheme 1b). The key to this multicomponent transformation hinges upon the discovery of a combined strong Brønsted acid system comprised of a chiral disulfonimide and 2-carboxyphenylboronic acid.

## Results and Discussion

We commenced the desired one-pot transformation by conducting the model reaction between phenylglyoxal monohydrate (**1a**), 4-methoxyaniline (**2a**), and PhSO_2_CF_2_CHN_2_ (**3**, Ps-DFA). Initial screenings were focused on the evaluation of various chiral phosphoric acids that have proven effective in similar aza-Darzens reactions of diazo esters and trifluorodiazoethane [36–39]. Unfortunately, these endeavors resulted in either no conversion or no enantioselectivity at all. As arylboronic acids have been harnessed to enhance the Brønsted acidity in asymmetric organocatalysis in combination with chiral diols or chiral aminoalcohols [40–44], we envisioned that the simultaneous use of arylboronic acids and chiral Brønsted acids may bring about a complementary catalytic platform. Encouragingly, the targeted CF_2_-functionalized aziridine **4a** was obtained in up to 51% ee and high diastereoselectivity, albeit in a low yield (Table 1, entries 1 and 2). The difficulty in further improving the conversions might be ascribed to the limited Brønsted acidity of chiral phosphoric acids. Bearing this in mind, we then turned our attention to chiral disulfonimides developed by List, which have been established as a unique type of stronger Brønsted acids [45]. Putting it into practice, a range of BINOL-derived disulfonimides was used as the chiral additive in combination with 2-carboxyphenylboronic acid (**COOH-BA**) in the model reaction (Table 1, entries 3–8). We were pleased to find that **CDSI-4** gave the most promising result in terms of both yield and enantioselectivity (64% isolated yield with 73% ee, Table 1, entry 6). An examination on various arylboronic acids, solvent, temperature, and catalyst loadings resulted in no obvious improvement (Table 1, entries 9–15). Among them, the highest yield of **4a** was observed (81%, Table 1, entry 12), albeit with slightly reduced ee value. This enhancement in catalytic activity could be attributed to the increased Brønsted acidity when the strong electron-withdrawing trifluoromethyl group was placed on the benzene ring of the arylboronic acid. Removing the boronic acid from the reaction system leads to a dramatic decrease in both yield and enantiocontrol (Table 1, entry 16).

**Table 1:** Representative screening results of the asymmetric aziridination reaction of PhSO_2_CF_2_CHN_2_.^a^

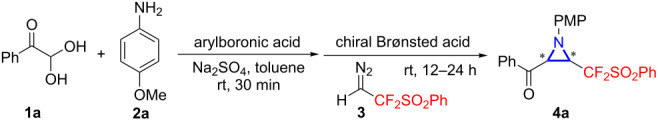				

entry	arylboronic acid (mol %)	chiral Brønsted acid (mol %)	yield of **4a** (%)^b^	ee (%) of **4a** and dr of crude mixture^c^

1	**COOH-BA** (8)	**CPA-1** (5)	24	51, 13:1
2	**COOH-BA** (8)	**CPA-2** (5)	28	25, 11:1
3	**COOH-BA** (8)	**CDSI-1** (5)	21	41, 19:1
4	**COOH-BA** (8)	**CDSI-2** (5)	50	41, 9:1
5^d^	**COOH-BA** (8)	**CDSI-3** (5)	16	60, 5:1
6^d^	**COOH-BA** (8)	**CDSI-4** (5)	64	73, 13:1
7	**COOH-BA** (8)	**CDSI-5** (5)	34	33, 10:1
8	**COOH-BA** (8)	**CDSI-6** (5)	47	52, 9:1
9	**OH-BA** (8)	**CDSI-4** (5)	63	68, 28:1
10^d^	**SO_3_H-BA** (8)	**CDSI-4** (5)	62	66, 16:1
11^d^	**NO_2_-BA** (8)	**CDSI-4** (5)	45	62, 16:1
12	**CF_3_-COOH-BA** (8)	**CDSI-4** (5)	81	67, 8:1
13^e^	**COOH-BA** (8)	**CDSI-4** (5)	60	47, 5:1
14^f^	**COOH-BA** (8)	**CDSI-4** (5)	trace	n.d.
15^d^	**COOH-BA** (8)	**CDSI-4** (10)	65	70, 12:1
16^d^	–	**CDSI-4** (5)	10	60, >20:1

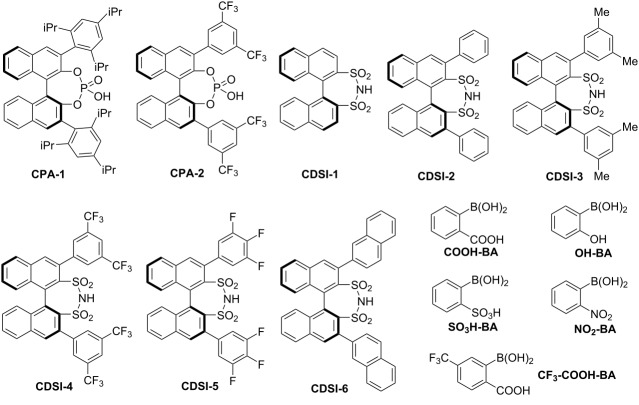
^a^General reaction conditions: **1a** (8 mg, 0.05 mmol, 1.0 equiv), **2a** (7 mg, 0.055 mmol), arylboronic acid (0.004 mmol), and Na_2_SO_4_ (40 mg) was stirred in toluene (1 mL) at rt for 30 min, then the chiral Brønsted acid (0.0025 mmol) and **3** (18 mg, 0.075 mmol) were added and the mixture was reacted at rt for 12 hours unless otherwise noted; ^b^yield of isolated product **4a** was given for entries labelled with d; hexafluorobenzene was used as an internal standard to determine the yield in other cases; ^c^ee of **4a** was determined by chiral HPLC analysis, and the dr of the crude reaction mixture was probed by ^19^F NMR analysis; ^d^0.3 mmol scale of reaction was conducted: **1a** (46 mg, 0.3 mmol, 1.0 equiv), **2a** (41 mg, 0.33 mmol), arylboronic acid (0.024 mmol), and Na_2_SO_4_ (200 mg) was stirred in toluene (2 mL) at rt for 30 min, then the chiral Brønsted acid (0.015 mmol) and **3** (105 mg, 0.45 mmol) were added and the mixture was reacted at rt for 12–24 hours; ^e^CH_2_Cl_2_ was used as the solvent; ^f^reaction was operated at 0 °C.

**Scheme 2:**
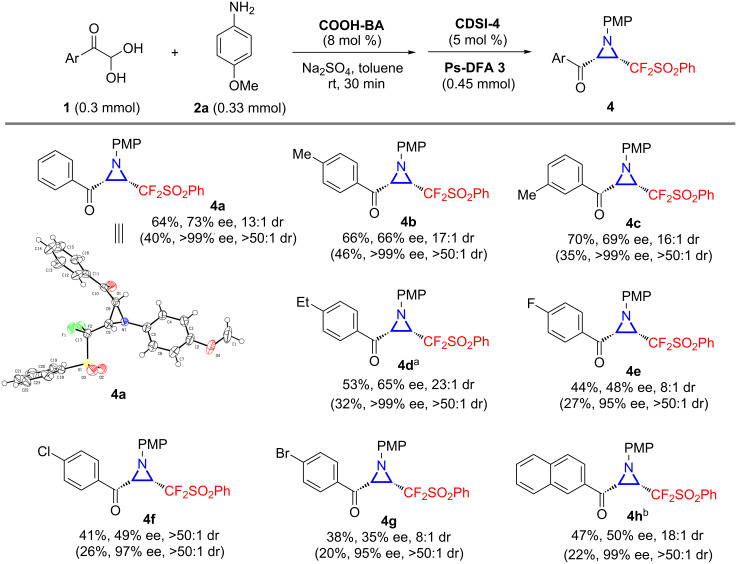
Substrate scope of chiral CF_2_-substituted aziridines from PhSO_2_CF_2_CHN_2_. General reaction conditions: Aryl glyoxal monohydrate (**1**, 0.3 mmol), **2a** (41 mg, 0.33 mmol), **COOH-BA** (4 mg, 0.024 mmol), and Na_2_SO_4_ (200 mg) were stirred in toluene (2 mL) at rt for 30 min, then **CDSI-4** (12 mg, 0.015 mmol) and Ps-DFA **3** (105 mg, 0.45 mmol) were added and the mixture was reacted at rt for 24 hours unless otherwise annotated. The yields are those of isolated products, and the dr was determined by ^19^F NMR analysis of the crude mixture. The results in parentheses are those of isolated products after the dissolution–filtration process: The corresponding CF_2_-functionalized aziridine **4** was dissolved in isopropanol (0.05–0.2 mL/mg) with the help of ultrasound, followed by filtration, and the obtained solution was concentrated to give **4** with increased ee and dr values. ^a^0.006 mmol of **COOH-BA** was employed. ^b^The reaction was operated at 45 °C for 24 h.

The challenge to further improve the enantioselectivity promoted us to search for other practical solutions. Considering the poor solubility of **4a** in organic solvents, a dissolution–filtration process with isopropanol was found to be workable for increasing the final ee value. This simple procedure could afford **4a** with excellent enantiopurity as a single diastereoisomer (>99% ee, >50:1 dr, Scheme 2). By the aid of the developed one-pot aza-Darzens reaction and dissolution–filtration operation, a series of optically-pure CF_2_-aziridines **4b**–**h** were furnished in moderate overall yields with uniformly excellent ee and dr values, including alkyl or halogen-substituted phenyl and 2-naphthyl ketones (Scheme 2). Unfortunately, phenylglyoxal monohydrates bearing strong electron-withdrawing groups were not compatible with the current conditions. X-ray analysis of aziridine **4a** confirmed the absolute configuration of the chiral centers, pointing at a *cis*-aziridination process [46].

**Scheme 3:**
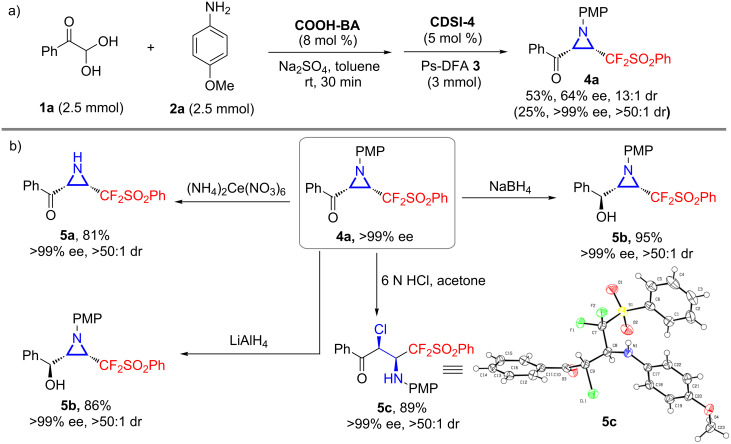
Scale-up experiment to **4a** and further synthetic transformations.

Scaled-up experiments with model substrate **1a** also proved to be feasible, delivering the chiral CF_2_-aziridine **4a** with comparable results (Scheme 3a). The 4-methoxyphenyl group of **4a** was cleaved smoothly with ceric ammonium nitrate, giving the free aziridine **5a** in 81% yield while maintaining the ee value. The reduction of the carbonyl moiety with either NaBH_4_ or LiAlH_4_ produced hydroxy-substituted CF_2_-functionalized aziridine **5b** in excellent yield with exclusive diastereoselectivity [47]. Furthermore, the ring-opening of **4a** under acidic conditions underwent well and gave rise to CF_2_-functionalized α-chloro-β-amino ketone **5c** in 89% yield with >99% ee and >50:1 dr (confirmed by X-ray spectroscopy) [46].

## Conclusion

In summary, an array of chiral CF_2_-functionalized aziridines was constructed from in situ-formed aldimines and difluorodiazoetyl phenyl sulfone under mild conditions by a combined strong Brønsted acid system consisting of chiral disulfonimide and 2-carboxyphenylboronic acid. The optical purity of the obtained CF_2_-substituted aziridines could be further improved by a practical dissolution–filtration procedure. Substrate expansion and mechanistic investigation are underway and will be reported in due course.

## Experimental

**General procedure for the preparation of chiral CF_2_-functionalized aziridines 4**: To a 25 mL Schlenk tube equipped with a stirring bar were added 2,2-dihydroxy-1-arylethan-1-one (**1**, 0.3 mmol, 1 equiv), 4-methoxyaniline (**2a**, 40.6 mg, 0.33 mmol), 2-boronobenzoic acid (**COOH-BA**, 3.98 mg, 0.024 mmol), anhydrous Na_2_SO_4_ (200 mg) and toluene (1 mL) at room temperature under an argon atmosphere. After reacting for 30 minutes at room temperature, ((2-diazo-1,1-difluoroethyl)sulfonyl)benzene (Ps-DFA **3**, 104.5 mg, 77.4 uL, 0.45 mmol) was added with a micro syringe and **CDSI-4** (12.3 mg, 0.015 mmol) in toluene (1 mL) was added dropwise. The reaction was allowed to stir for 24 hours at room temperature under an argon atmosphere until the consumption of substrates was completed (as monitored by TLC). The reaction mixture was quenched with saturated aq NaHCO_3_ and extracted with ethyl acetate three times. The combined organic layer was washed with water and brine, and then dried over anhydrous Na_2_SO_4_, filtered and evaporated under vacuum. The residue was purified by neutral alumina column chromatography (eluting with dichloromethane/petroleum ether) to give CF_2_-substituted aziridine **4**. The enantiomeric excess was determined by chiral HPLC analysis. See Supporting Information File 1 for the dissolution–filtration procedure for each compound.

## Supporting Information

File 1 Experimental procedures, compound characterization, NMR spectra of all new compounds, and HPLC traces.

File 2 X-ray data for compound **4a**.

File 3 X-ray data for compound **5c**.
