# Supplementary material for: Asymmetric synthesis of CF2-functionalized aziridines by combined strong Brønsted acid catalysis
Source: Beilstein J Org Chem. 2020 Apr 7;16:638–44. doi: 10.3762/bjoc.16.60 (PMC7155912; doi:10.3762/bjoc.16.60)
Supplement: File 1 — Experimental procedures, compound characterization, NMR spectra of all new compounds, and HPLC traces. [file Beilstein_J_Org_Chem-16-638-s001.pdf]

BEILSTEIN JOURNAL OF ORGANIC CHEMISTRY

Supporting Information

for

Asymmetric synthesis of CF_2-functionalized aziridines by combined strong Brønsted acid catalysis

Xing-Fa Tan, Fa-Guang Zhang and Jun-An Ma

Beilstein J. Org. Chem. **2020,** *16,* 638–644. doi:10.3762/bjoc.16.60

Experimental procedures, compound characterization, NMR spectra of all new compounds, and HPLC traces

License and Terms: This is a supporting information file under the terms of the Creative Commons Attribution License (http://creativecommons.org/licenses/by/4.0). Please note that the reuse, redistribution and reproduction in particular requires that the authors and source are credited.
The license is subject to the *Beilstein Journal of Organic Chemistry* terms and conditions: (https://www.beilstein-journals.org/bjoc)

Contents

1 General information S3

2 Experimental Section and HPLC charts for chiral compounds 4 and 5 ....S4

2.1 Typical procedure I: preparation of racemic *cis*-CF_2-aziridine 4 ....S4

2.2 Typical procedure II: preparation of chiral *cis*-CF_2-aziridine 4 ....S4

2.3 Preparation of compound 5a..S14

2.4 Preparation of compound 5b..S15

2.5 Preparation of compound 5c ………………………………………… S17

3 References...S18

4 NMR spectra of all the new compounds S19

5 X-ray crystallographic data…………………………………………………… S36

5.1 X-ray crystallographic structures for compound 4a. Crystal data have been deposited to CCDC, number 1983642……………….……………..………..S36

5.2 X-ray crystallographic structures for compound 5c. Crystal data have been deposited to CCDC, number 1983643…………………………..……...S37

1 General information

^{1}H, ^{13}C and ^{19}F NMR spectra were recorded on a Bruker AV 400 MHz instrument at 400 MHz (^{1}H NMR), 101 MHz (^{13}C NMR), as well as 377 MHz (^{19}F NMR), or a Bruker AV at 500 MHz instrument at 500 MHz (^{1}H NMR). Chemical shifts are reported in ppm down field from the internal standard Me_4Si and external CCl_3F, respectively. The multiplicity is indicated as follows: s (singlet), d (doublet), t (triplet), q (quartet), m (multiplet), dd (doublet of doublets), ddd (doublet of doublets of doublets), tt (triplet of triplets), dt (triplet of doublets), ddt (triplet of doublets of doublets). Coupling constants are reported in Hertz (Hz). MS were recorded on a VG ZABHS spectrometer with an ESI source. High resolution mass spectrometry (HRMS) spectra were obtained on a Bruker microTOF-QII instrument. Optical rotations were determined using an Autopol IV-T. HPLC analyses were carried out on a HewlettPackard Model HP 1200 instrument. X-ray structural analysis was conducted on a Bruker APEX-II CCD instrument.

Materials: Tetrahydrofuran (THF), diethyl ether (Et_2O) and toluene (Tol) were distilled from sodium/benzophenone prior to use; CH_2Cl_2 (DCM) and $ClCH_2CH_2Cl$ (DCE) were distilled from CaH_2. MeCN was distilled from P_2O_5; All purchased reagents were used without further purification. Analytical thin layer chromatography was performed on 0.20 mm Qingdao Haiyang silica gel plates. Silica gel (200-300 mesh) (from Qingdao Haiyang Chem. Company, Ltd.) was used for flash chromatography. ((2-diazo-1,1-difluoroethyl)sulfonyl)benzene (Ps-DFA) were prepared according to the reported procedure.[1] Chiral disulfonimide catalysts **CDSI-1**, **CDSI-2**, **CDSI-3**, **CDSI-4**, **CDSI-5**, **CDSI-6** were synthesized according to the known procedures.[2] 2-Borono-4-(trifluoromethyl)benzoic acid (**CF_3-COOH-BA**) used in this work is a known compound and was prepared according to literature procedures.[3] $Mg(TMP)_2$[4] and 2,2-dihydroxy-1-arylethan-1-one[5] were both prepared according to the literature.

2 Experimental section and HPLC charts for the chiral compounds 4 and 5

2.1 Typical procedure I: preparation of racemic *cis*-CF_2-aziridine 4

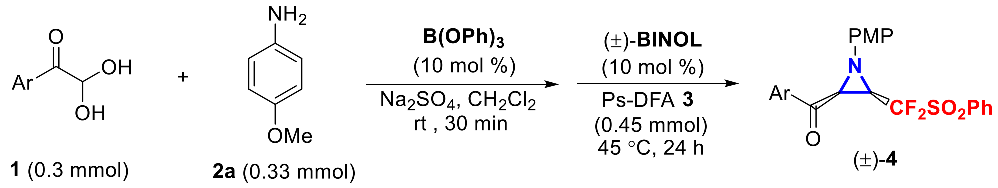

To a 25 mL Schlenk tube equipped with a reflux condenser and an argon balloon at the top of the condenser through a rubber septum was added 2,2-dihydroxy-1-arylethan-1-one (**1**, 0.3 mmol, 1 equiv), 4-methoxyaniline (**2a**, 40.6 mg, 0.33 mmol), triphenyl borate $B(OPh)_3$ (8.7 mg, 0.03 mmol), anhydrous Na_2SO_4 (200 mg) and CH_2Cl_2 (1 mL) at room temperature under an argon atmosphere. After reacting for 30 minutes at room temperature, the ((2-diazo-1,1-difluoroethyl)sulfonyl)benzene (Ps-DFA **3**, 104.5 mg, 77.4 μL, 0.45 mmol) was added with a micro syringe and racemic 1,1'-bi-2-naphthol (**BINOL**, 8.6 mg, 0.03 mmol) in CH_2Cl_2 (1 mL) was added dropwise. The reaction was allowed to stir for 24 hours at 45 °C under an argon atmosphere until the consumption of substrates was completed (monitored by TLC). The reaction mixture was extracted with CH_2Cl_2 three times. The combined organic layer was washed with water and brine, and then dried over anhydrous Na_2SO_4, filtered and evaporated under vacuum. The residue was purified by neutral alumina column chromatography (eluting with dichloromethane/petroleum ether) to give racemic *cis*-CF_2-aziridine **4**.

2.2 Typical procedure II: preparation of chiral *cis*-CF_2-aziridine 4

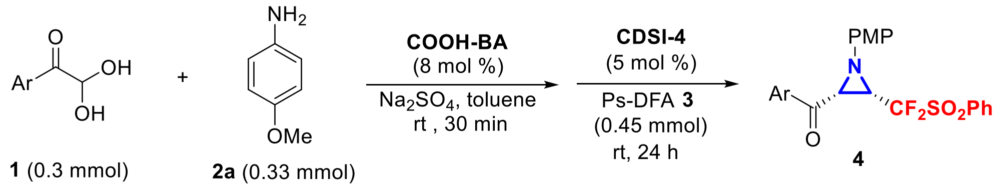

To a 25 mL Schlenk tube equipped with a stirring bar were added 2,2-dihydroxy-1-arylethan-1-one (**1**, 0.3 mmol, 1 equiv), 4-methoxyaniline (**2a**, 40.6 mg, 0.33 mmol), 2-boronobenzoic acid (**COOH-BA**, 3.98 mg, 0.024 mmol), anhydrous Na_2SO_4 (200 mg) and toluene (1 mL) at room temperature under argon atmosphere. After reacting for 30 minutes at room temperature, the ((2-diazo-1,1-difluoroethyl)sulfonyl)benzene (Ps-DFA, **3**, 104.5 mg, 77.4 uL, 0.45 mmol) was added with a micro syringe and **CDSI-4** (12.3 mg, 0.015 mmol) in toluene (1 mL) was added dropwise. The reaction was allowed to stir for 24 hours at room temperature under an argon atmosphere until the consumption of substrates was completed (monitored by TLC). The reaction mixture was quenched with saturated aq. $NaHCO_3$ and extracted with ethyl acetate three times. The combined organic layer was washed with water and brine, and then dried over anhydrous Na_2SO_4, filtered and evaporated under vacuum. The residue was purified by neutral alumina column chromatography (eluting with dichloromethane/petroleum ether) to give *cis*-CF_2-aziridine **4**. The enantiomeric excess was determined by chiral HPLC analysis.

((2*R*,3*S*)-3-(Difluoro(phenylsulfonyl)methyl)-1-(4-methoxyphenyl)aziridin-2-yl)(phenyl)methanone (4a)

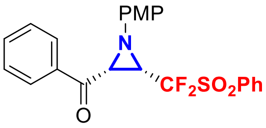

The *cis*-CF_2-aziridine **4a** was obtained as white solid after neutral alumina column chromatography (petroleum ether/dichloromethane = 2:1), 85 mg, 64% yield, 73% ee; M.p. 175.8-176.7 °C; R_f = 0.3 (dichloromethane/ petroleum ether = 3:1); **4a** with 73% ee (85 mg) was dissolved in an appropriate amount of isopropanol (0.15 mL/mg) with ultrasound for 3 minutes, followed by filtration, and the obtained solution was concentrated to give **4a** with more than 99% ee; repeating the above operation led to 99% ee analyzed by HPLC. The obtained solution was combined and concentrated to give **4a** (40% yield, >99% ee).

^{1}H NMR (400 MHz, $CDCl_3$) δ 8.20 – 8.05 (m, 2H), 7.93 (d, *J* = 7.7 Hz, 2H), 7.71 (t, *J* = 7.5 Hz, 1H), 7.57 (dt, *J* = 15.6, 7.6 Hz, 3H), 7.46 (t, *J* = 7.7 Hz, 2H), 7.25 – 7.17 (m, 2H), 6.90 – 6.81 (m, 2H), 3.77 (s, 3H), 3.67 (d, *J* = 6.6 Hz, 1H), 3.57 (dt, *J* = 16.0, 6.1 Hz, 1H). **^{13}C NMR** (101 MHz, $CDCl_3$) δ 190.6, 156.7, 143.8, 135.8, 135.5, 134.1, 132.0, 130.9, 129.5, 129.0, 128.8, 121.2, 120.3 (dd, J_{C-F} = 291.5, 283.7 Hz), 114.7, 55.6, 45.5, 42.2 (dd, J_{C-F} = 30.2, 20.5 Hz). **^{19}F NMR** (377 MHz, $CDCl_3$) δ -102.81 (dd, *J* = 237.2, 5.7 Hz), -106.31 (dd, *J* = 237.2, 16.0 Hz). **HRMS** (ESI) m/z calcd for $C_{23}H_{20}NO_4F_2S$ $[M+H]^+$: 444.1081, found: 444.1081; $[\alpha]_D^{20}$ = 67.2 (c 1.0, CH_2Cl_2), >99% ee; **HPLC** (column, Daicel Chirapak IC, n-Hexane/i-PrOH = 70/30, flow rate 1.0 mL/min, 20 °C detection UV 254 nm) t_R of major isomer 38.1 min, t_R of minor isomer 26.3 min.

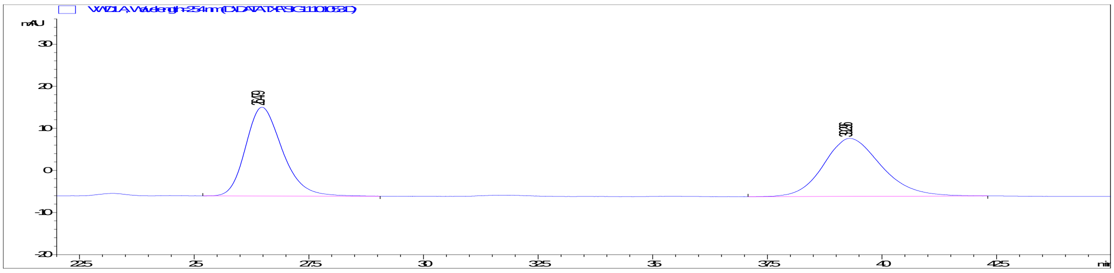

Peak #	Ret.Time (min)	Area mAU *S	Height (mAU)	Width (min)	Symmetry Factor	Area (%)
1	26.479	1173.6	21.1	0.8439	0.762	49.265
2	39.296	1208.6	13.8	1.1724	0.808	50.735

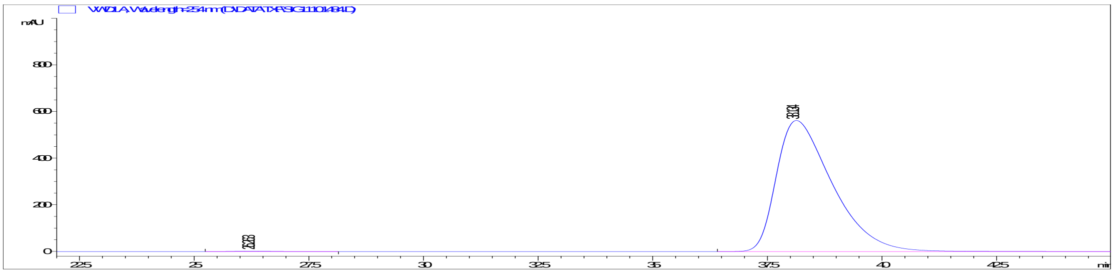

Peak #	Ret.Time (min)	Area mAU *S	Height (mAU)	Width (min)	Symmetry Factor	Area (%)
1	26.263	85.3	1.5	0.6986	0.868	0.178
2	38.134	47676	561.9	1.2956	0.52	99.822

((2*R*,3*S*)-3-(difluoro(phenylsulfonyl)methyl)-1-(4-methoxyphenyl)aziridin-2-yl)(p-tolyl)methanone (4b)

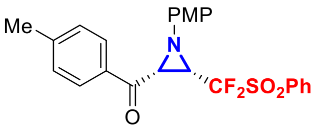

The *cis*-CF_2-aziridine **4b** was obtained as white solid after neutral alumina column chromatography (petroleum ether/dichloromethane = 2:1), 90 mg, 66% yield, 66% ee; M.p. 154.3-154.9 °C; R_f = 0.2 (dichloromethane/ petroleum ether = 3:1); **4b** with 66% ee (90 mg) was dissolved in an appropriate amount of isopropanol (0.1 mL/mg) with ultrasound for 3 minutes, followed by filtration, and the obtained solution was concentrated to give **4b** with more than 99% ee. Repeating the above operation led to 99% ee analyzed by HPLC; The obtained solution was combined and concentrated to give **4b** (46% yield, >99% ee).

^{1}H NMR (400 MHz, $CDCl_3$) δ 8.04 (d, *J* = 8.2 Hz, 2H), 7.95 (d, *J* = 7.8 Hz, 2H), 7.73 (t, *J* = 7.5 Hz, 1H), 7.57 (t, *J* = 7.8 Hz, 2H), 7.30 – 7.26 (m, 2H), 7.24 (t, *J* = 6.1 Hz, 2H), 6.87 (d, *J* = 8.9 Hz, 2H), 3.79 (s, 3H), 3.67 (d, *J* = 6.6 Hz, 1H), 3.57 (dt, *J* = 16.2, 6.1 Hz, 1H), 2.41 (s, 3H). **^{13}C NMR** (101 MHz, $CDCl_3$) δ 190.1, 156.7, 145.1, 143.9, 135.8, 133.0, 132.0, 130.9, 129.4, 129.1, 121.2, 120.3 (dd, $J_{C\text{-}F}$ = 291.6, 283.7 Hz), 114.7, 55.6, 45.5, 42.1 (dd, $J_{C\text{-}F}$ = 30.3, 20.4 Hz), 21.8. **^{19}F NMR** (377 MHz, $CDCl_3$) δ -102.84 (dd, *J* = 237.2, 4.2 Hz), -106.52 (dd, *J* = 237.1, 16.2 Hz). **HRMS** (ESI) m/z calcd for $C_{24}H_{22}NO_4F_2S$ $[M+H]^+$: 458.1238, found: 458.1237; $[\alpha]_D^{20}$ = 91.4 (c 1.0, CH_2Cl_2), >99% ee; **HPLC** (column, Daicel Chirapak IC, n-Hexane/i-PrOH = 70/30, flow rate 1.0 mL/min, 20 °C detection UV 254 nm) t_R of major isomer 48.8 min, t_R of minor isomer 30.2 min.

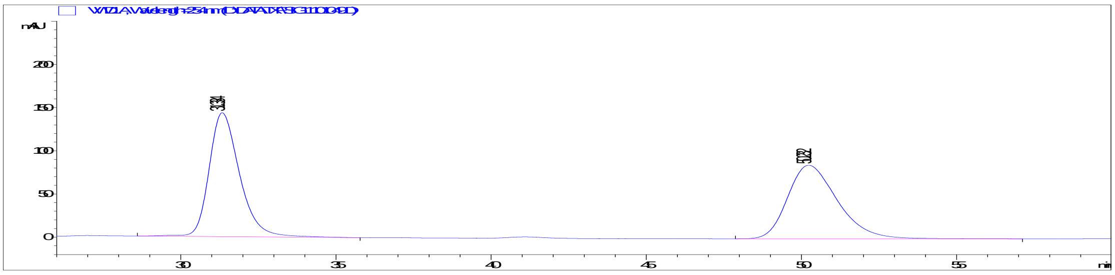

Peak #	Ret.Time (min)	Area mAU *S	Height (mAU)	Width (min)	Symmetry Factor	Area (%)
1	31.334	9801.6	143.4	1.048	0.715	50.756
2	50.232	9509.5	85.1	1.659	0.701	49.244

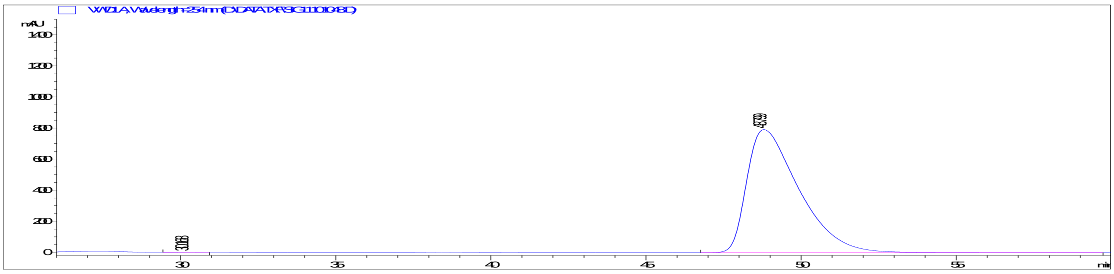

Peak #	Ret.Time (min)	Area mAU *S	Height (mAU)	Width (min)	Symmetry Factor	Area (%)
1	30.168	48.8	1.1	0.6399	1.076	0.053
2	48.799	92497.9	792.1	1.7643	0.455	99.947

((2*R*,3*S*)-3-(Difluoro(phenylsulfonyl)methyl)-1-(4-methoxyphenyl)aziridin-2-yl)(m-tolyl)methanone (4c)

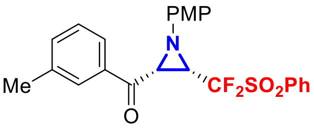

The *cis*-CF_2-aziridine **4c** was obtained as white solid after neutral alumina column chromatography (petroleum ether/dichloromethane = 2:1), 94 mg, 70% yield, 69% ee; M.p. 157.0-158.0 °C; R_f = 0.3 (dichloromethane/ petroleum ether = 3:1); **4c** with 69% ee (94 mg) was dissolved in an appropriate amount of isopropanol (0.15 mL/mg) with ultrasound for 3 minutes, followed by filtration, and the obtained solid with increased ee; Repeating the above operation until the obtained solid was more than 99% ee analyzed by HPLC; The obtained solid **4c** (35% yield, >99% ee).

^{1}H NMR (400 MHz, $CDCl_3$) δ 7.92 (d, *J* = 8.1 Hz, 4H), 7.69 (t, *J* = 7.5 Hz, 1H), 7.53 (t, *J* = 7.8 Hz, 2H), 7.38 (d, *J* = 7.5 Hz, 1H), 7.33 (t, *J* = 7.5 Hz, 1H), 7.20 (d, *J* = 8.8 Hz, 2H), 6.84 (d, *J* = 8.9 Hz, 2H), 3.75 (s, 3H), 3.68 (d, *J* = 6.6 Hz, 1H), 3.56 (dt, *J* = 16.3, 6.1 Hz, 1H), 2.37 (s, 3H). **^{13}C NMR** (101 MHz, $CDCl_3$) δ 190.5, 156.5, 143.8, 138.5, 135.7, 135.3, 134.7, 131.8, 130.7, 129.3, 129.1, 128.5, 126.0, 121.0, 120.2 (dd, $J_{C\text{-}F}$ = 291.6, 283.4 Hz), 114.5, 55.4, 45.4, 42.0 (dd, $J_{C\text{-}F}$ = 30.4, 20.2 Hz), 21.2. **^{19}F NMR** (377 MHz, $CDCl_3$) δ -102.66 (dt, *J* = 237.2, 6.0 Hz), -106.51 (dt, *J* = 237.2, 15.9 Hz). **HRMS** (ESI) m/z calcd for $C_{24}H_{22}NO_4F_2S$ $[M+H]^+$: 458.1238, found: 458.1237; $[\alpha]_D^{20}$ = 80.8 (c 1.0, CH_2Cl_2), 99% ee; **HPLC** (column, Daicel Chirapak IC, n-Hexane/i-PrOH = 70/30, flow rate 1.0 mL/min, 20 °C detection UV 254 nm) t_R of major isomer 40.4 min, t_R of minor isomer 27.1 min.

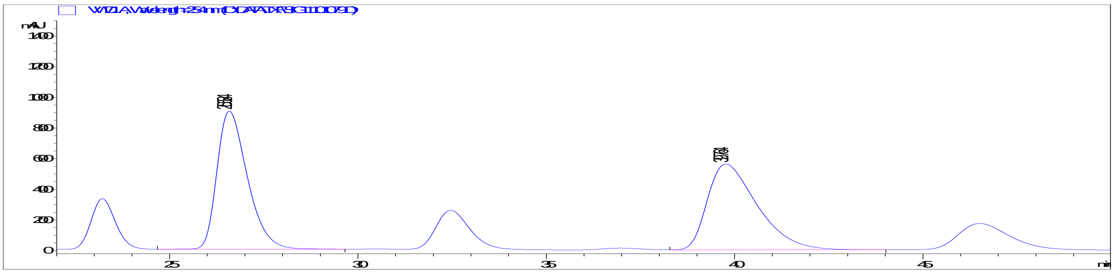

Peak #	Ret.Time (min)	Area mAU *S	Height (mAU)	Width (min)	Symmetry Factor	Area (%)
1	26.584	50779.4	902.6	0.866	0.647	49.595
2	39.764	51609.3	560.9	1.4171	0.546	50.405

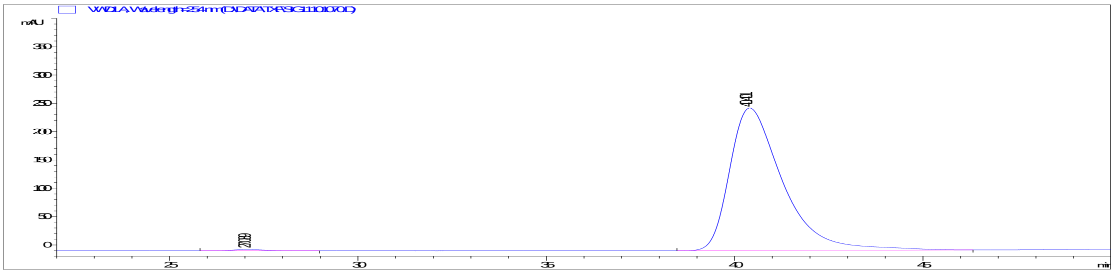

Peak #	Ret.Time (min)	Area mAU *S	Height (mAU)	Width (min)	Symmetry Factor	Area (%)
1	27.089	136.4	2.2	0.752	0.886	0.551
2	40.401	24617.7	251.7	1.4701	0.565	99.449

((2*R*,3*S*)-3-(Difluoro(phenylsulfonyl)methyl)-1-(4-methoxyphenyl)aziridin-2-yl)(4-ethylphenyl)methanone (4d)

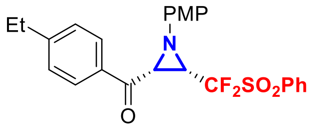

The *cis*-CF_2-aziridine **4d** was obtained as white solid after neutral alumina column chromatography (petroleum ether/dichloromethane = 2:1), 75 mg, 53% yield, 65% ee; M.p. 160.8-161.6 °C; R_f = 0.25 (dichloromethane/ petroleum ether = 3:1); **4d** with 65% ee (75 mg) was dissolved in an appropriate amount of isopropanol (0.05 mL/mg) with ultrasound for 3 minutes, followed by filtration, and the obtained solution was concentrated to give **4d** with more than 99% ee; Repeating the above operation led to 99% ee analyzed by HPLC; The obtained solution was combined and concentrated to give **4d** (32% yield, >99% ee).

^{1}H NMR (400 MHz, $CDCl_3$) δ 8.05 (d, *J* = 8.2 Hz, 2H), 7.94 (d, *J* = 7.8 Hz, 2H), 7.71 (t, *J* = 7.5 Hz, 1H), 7.55 (t, *J* = 7.9 Hz, 2H), 7.28 (d, *J* = 8.2 Hz, 2H), 7.25 – 7.17 (m, 2H), 6.91 – 6.80 (m, 2H), 3.77 (s, 3H), 3.65 (d, *J* = 6.6 Hz, 1H), 3.61 – 3.51 (m, 1H), 2.69 (q, *J* = 7.6 Hz, 2H), 1.24 (t, *J* = 7.6 Hz, 3H). **^{13}C NMR** (101 MHz, $CDCl_3$) δ 190.1, 156.7, 151.2, 144.0, 135.8, 133.3, 132.1, 131.0, 129.5, 129.2, 128.3, 121.2, 120.3 (dd, $J_{C\text{-}F}$ = 291.6, 283.6 Hz), 114.7, 55.7, 45.5, 42.1 (dd, $J_{C\text{-}F}$ = 30.4, 20.2 Hz), 29.1, 15.1. **^{19}F NMR** (377 MHz, $CDCl_3$) δ -102.71 (dd, *J* = 237.2, 5.1 Hz), -106.61 (dd, *J* = 237.2, 16.3 Hz). **HRMS** (ESI) m/z calcd for $C_{25}H_{24}NO_4F_2S$ $[M+H]^+$: 472.1394, found: 472.1395; $[\alpha]_D^{20}$ = 76.8 (c 1.0, CH_2Cl_2), 99% ee; **HPLC** (column, Daicel Chirapak IC, n-Hexane/i-PrOH = 70/30, flow rate 1.0 mL/min, 20 °C detection UV 254 nm) t_R of major isomer 44.0 min, t_R of minor isomer 30.1 min.

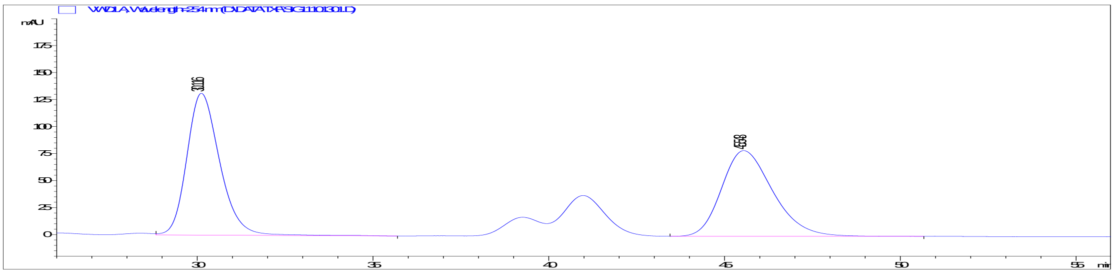

Peak #	Ret.Time (min)	Area mAU *S	Height (mAU)	Width (min)	Symmetry Factor	Area (%)
1	30.116	8571.6	131.4	1	0.74	51.615
2	45.543	8035.2	79.3	1.5426	0.727	48.385

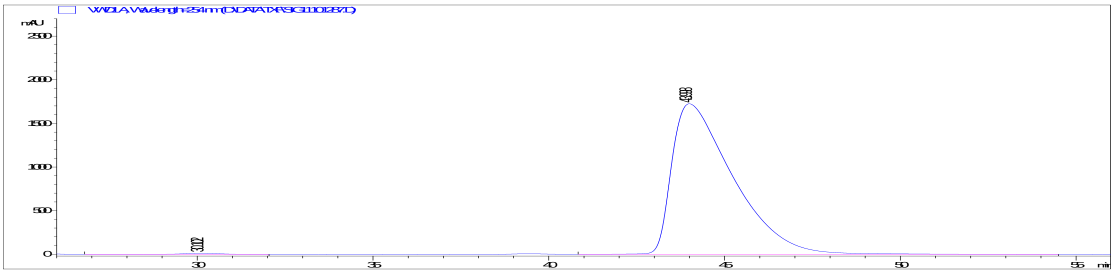

Peak #	Ret.Time (min)	Area mAU *S	Height (mAU)	Width (min)	Symmetry Factor	Area (%)
1	30.102	908.9	10.8	1.2291	1.282	0.434
2	43.998	208671.1	1725.6	1.7894	0.374	99.566

((2*R*,3*S*)-3-(Difluoro(phenylsulfonyl)methyl)-1-(4-methoxyphenyl)aziridin-2-yl)(4-fluorophenyl)methanone (4e)

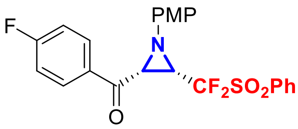

The *cis*-CF_2-aziridine **4e** was obtained as white solid after neutral alumina column chromatography (petroleum ether/dichloromethane = 2:1), 61 mg, 44% yield, 48% ee; M.p. 150.1-150.8 °C; R_f = 0.4 (dichloromethane/ petroleum ether = 3:1); **4e** with 48% ee (61 mg) was dissolved in an appropriate amount of isopropanol (0.05 mL/mg) with ultrasound for 3 minutes, followed by filtration, and the obtained solution was concentrated to give **4e** with more than 90% ee; Repeating the above operation led to 90% ee analyzed by HPLC; The obtained solution was combined and concentrated to give **4e** (27% yield, 95% ee determined by HPLC).

^{1}H NMR (400 MHz, $CDCl_3$) δ 8.23 – 8.12 (m, 2H), 7.94 (d, *J* = 7.7 Hz, 2H), 7.74 (t, *J* = 7.5 Hz, 1H), 7.57 (t, *J* = 7.9 Hz, 2H), 7.23 – 7.18 (m, 2H), 7.17 – 7.10 (m, 2H), 6.90 – 6.81 (m, 2H), 3.79 (s, 3H), 3.62 (d, *J* = 6.5 Hz, 1H), 3.55 (dt, *J* = 15.3, 6.4 Hz, 1H).

^{13}C NMR (101 MHz, $CDCl_3$) δ 189.3, 166.3 (d, J_{C-F} = 254.9 Hz), 156.9, 143.7, 135.9, 132.1, 132.0, 131.9 (d, J_{C-F} = 9.5 Hz), 131.0, 129.5, 121.2, 120.3 (dd, J_{C-F} = 291.1,

284.0 Hz), 116.0 (d, $J_{C\text{-}F}$ = 21.9 Hz), 114.9, 55.7, 45.4, 42.2 (dd, $J_{C\text{-}F}$ = 29.7, 20.8 Hz). **^{19}F NMR** (377 MHz, $CDCl_3$) δ -103.21 (tt, J = 8.4, 5.4 Hz), -103.35 (dd, J = 237.4, 6.2 Hz), -106.00 (dd, J = 237.4, 15.3 Hz). **HRMS** (ESI) m/z calcd for $C_{23}H_{19}NO_4F_3S$ $[M+H]^+$: 462.0987, found: 462.0980; $[\alpha]_D^{20}$ = 61.6 (c 1.0, CH_2Cl_2), 95% ee; **HPLC** (column, Daicel Chirapak IC, n-Hexane/i-PrOH = 70/30, flow rate 1.0 mL/min, 20 °C detection UV 254 nm) t_R of major isomer 31.2 min, t_R of minor isomer 22.4 min.

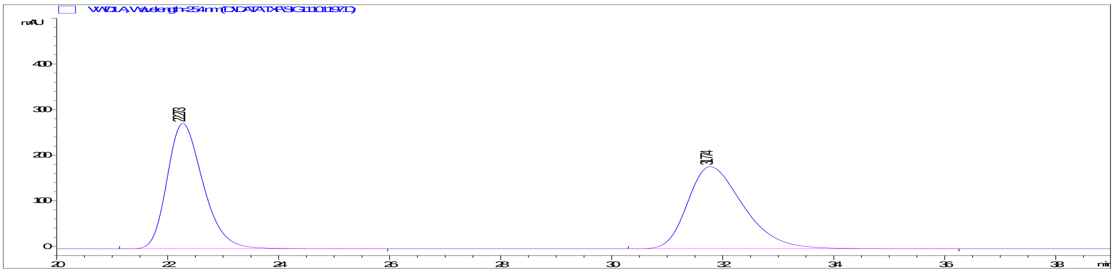

Peak #	Ret.Time (min)	Area mAU *S	Height (mAU)	Width (min)	Symmetry Factor	Area (%)
1	22.273	12384.4	274.8	0.6946	0.723	49.578
2	31.774	12595.5	180.2	1.0574	0.645	50.422

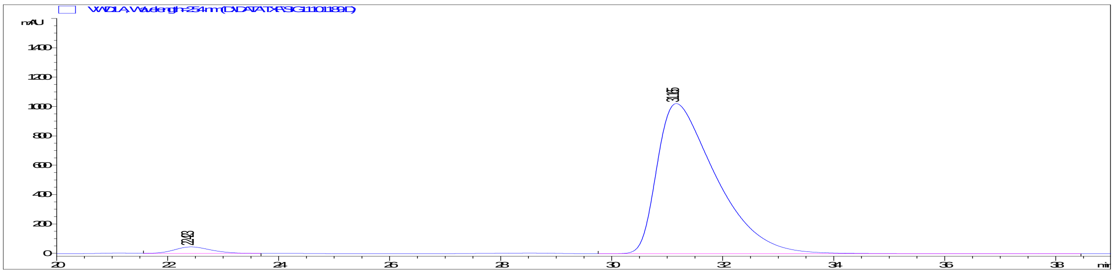

Peak #	Ret.Time (min)	Area mAU *S	Height (mAU)	Width (min)	Symmetry Factor	Area (%)
1	22.423	2067.3	44.6	0.7123	0.834	2.667
2	31.165	75454.7	1022.4	1.1056	0.449	97.333

(4-Chlorophenyl)((2*R*,3*S*)-3-(difluoro(phenylsulfonyl)methyl)-1-(4-methoxyphenyl)aziridin-2-yl)methanone (4f)

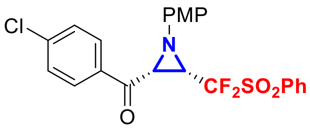

The *cis*-CF_2-aziridine **4f** was obtained as white solid after neutral alumina column chromatography (petroleum ether/dichloromethane = 2:1), 59 mg, 41% yield, 49% ee; M.p. 134.9-135.8 °C; R_f = 0.4 (dichloromethane/ petroleum ether = 3:1); **4f** with 49% ee (59 mg) was dissolved in an appropriate amount of isopropanol (0.05 mL/mg) with ultrasound for 3 minutes, followed by filtration, and the obtained solution was concentrated to give **4f** with more than 90% ee; Repeating

the above operation led to 90% ee analyzed by HPLC; The obtained solution was combined and concentrated to give **4f** (26% yield, 97% ee determined by HPLC).

^{1}H NMR (400 MHz, $CDCl_3$) δ 8.08 (d, J = 8.6 Hz, 2H), 7.94 (d, J = 7.8 Hz, 2H), 7.74 (t, J = 7.5 Hz, 1H), 7.58 (t, J = 7.8 Hz, 2H), 7.44 (d, J = 8.5 Hz, 2H), 7.20 (d, J = 8.9 Hz, 2H), 6.87 (d, J = 8.9 Hz, 2H), 3.79 (s, 3H), 3.61 (d, J = 6.5 Hz, 1H), 3.55 (dt, J = 15.0, 6.4 Hz, 1H). **^{13}C NMR** (101 MHz, $CDCl_3$) δ 189.8, 156.9, 143.6, 140.7, 136.0, 133.9, 132.0, 131.0, 130.5, 129.6, 129.2, 121.2, 120.3 (dd, J_{C-F} = 291.1, 284.2 Hz), 114.9, 55.7, 45.4, 42.3 (dd, J_{C-F} = 29.6, 20.9 Hz). **^{19}F NMR** (377 MHz, $CDCl_3$) δ -103.35 (dd, J = 237.4, 6.3 Hz), -105.87 (dd, J = 237.4, 15.1 Hz). **HRMS** (ESI) m/z calcd for $C_{23}H_{19}NO_4F_2SCl$ $[M+H]^+$: 478.0691, found: 478.0693; $[\alpha]_D^{20}$ = 58.4 (c 1.0, CH_2Cl_2), 97% ee; **HPLC** (column, Daicel Chirapak IC, n-Hexane/i-PrOH = 70/30, flow rate 1.0 mL/min, 20 ^{o}C detection UV 254 nm) t_R of major isomer 29.9 min, t_R of minor isomer 22.7 min.

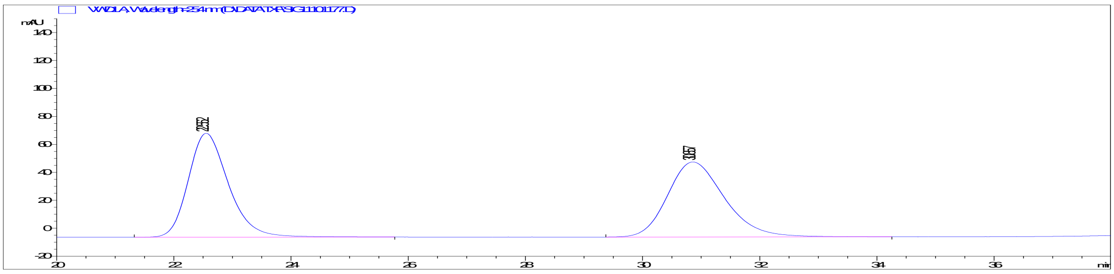

Peak #	Ret.Time (min)	Area mAU *S	Height (mAU)	Width (min)	Symmetry Factor	Area (%)
1	22.552	3533.5	74.3	0.7295	0.751	49.357
2	30.857	3625.6	53.7	1.0384	0.752	50.643

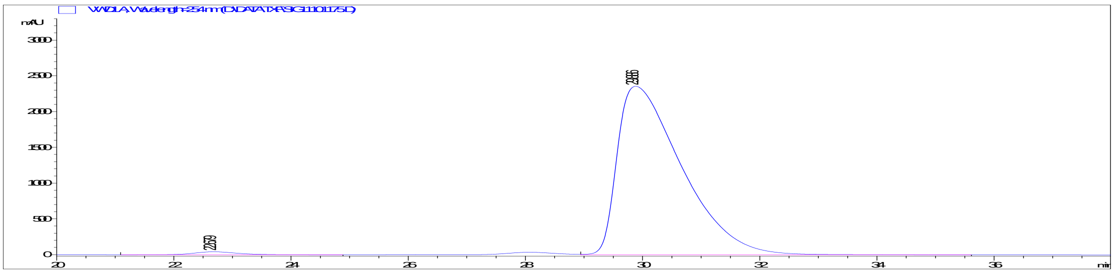

Peak #	Ret.Time (min)	Area mAU *S	Height (mAU)	Width (min)	Symmetry Factor	Area (%)
1	22.679	2417.5	43.1	0.8233	0.769	1.324
2	29.886	180194.8	2357.7	1.1538	0.377	98.676

(4-Bromophenyl)((2*R*,3*S*)-3-(difluoro(phenylsulfonyl)methyl)-1-(4-methoxyphenyl)aziridin-2-yl)methanone (4g)

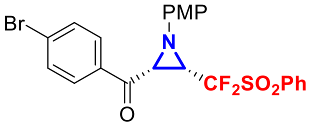

The *cis*-CF_2-aziridine **4g** was obtained as white solid after neutral alumina column chromatography (petroleum ether/dichloromethane = 2:1), 60 mg, 38% yield, 35% ee; M.p. 146.1-146.9 °C; R_f = 0.4 (dichloromethane/ petroleum ether = 3:1); **4g** with 35% ee (60 mg) was dissolved in an appropriate amount of isopropanol (0.2 mL/mg) with ultrasound for 3 minutes, followed by filtration, and the obtained solution was concentrated to give **4g** with more than 90% ee; Repeating the above operation led to 90% ee analyzed by HPLC; The obtained solution was combined and concentrated to give **4g** (20% yield, 95% ee determined by HPLC).

^{1}H NMR (400 MHz, $CDCl_3$) δ 8.00 (d, *J* = 8.5 Hz, 2H), 7.94 (d, *J* = 7.8 Hz, 2H), 7.74 (t, *J* = 7.5 Hz, 1H), 7.59 (dd, *J* = 17.1, 8.3 Hz, 4H), 7.23 – 7.15 (m, 2H), 6.87 (t, *J* = 6.0 Hz, 2H), 3.79 (s, 3H), 3.61 (d, *J* = 6.5 Hz, 1H), 3.55 (dt, *J* = 15.1, 6.4 Hz, 1H). **^{13}C NMR** (101 MHz, $CDCl_3$) δ 190.0, 156.9, 143.6, 135.9, 134.3, 132.2, 132.0, 131.0, 130.5, 129.6, 129.5, 121.2, 120.3 (dd, J_{C-F} = 291.1, 284.2 Hz), 114.9, 55.7, 45.4, 42.3 (dd, J_{C-F} = 29.7, 21.0 Hz). **^{19}F NMR** (377 MHz, $CDCl_3$) δ -103.34 (dd, *J* = 237.4, 6.3 Hz), -105.84 (dd, *J* = 237.4, 15.0 Hz). **HRMS** (ESI) m/z calcd for $C_{23}H_{19}NO_4F_2SBr$ $[M+H]^+$: 522.0186, found: 522.0184; $[\alpha]_D^{20}$ = 53.8 (c 1.0, CH_2Cl_2), 95% ee; **HPLC** (column, Daicel Chirapak IC, n-Hexane/i-PrOH = 70/30, flow rate 1.0 mL/min, 20 °C detection UV 254 nm) t_R of major isomer 33.2 min, t_R of minor isomer 24.8 min.

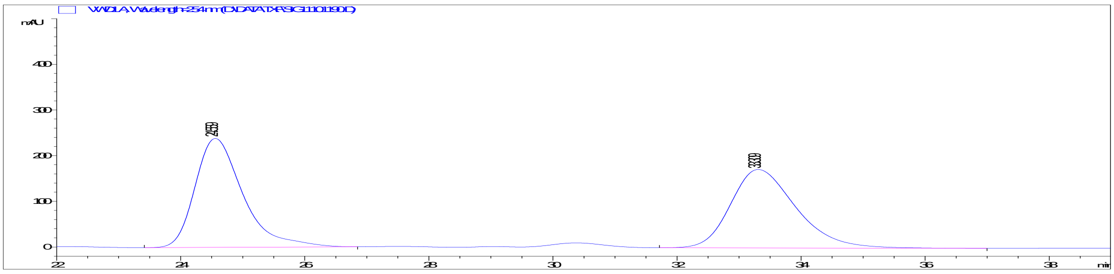

Peak #	Ret.Time (min)	Area mAU *S	Height (mAU)	Width (min)	Symmetry Factor	Area (%)
1	24.559	12743.6	238.3	0.8191	0.689	50.344
2	33.309	12569.3	171.7	1.128	0.7	49.656

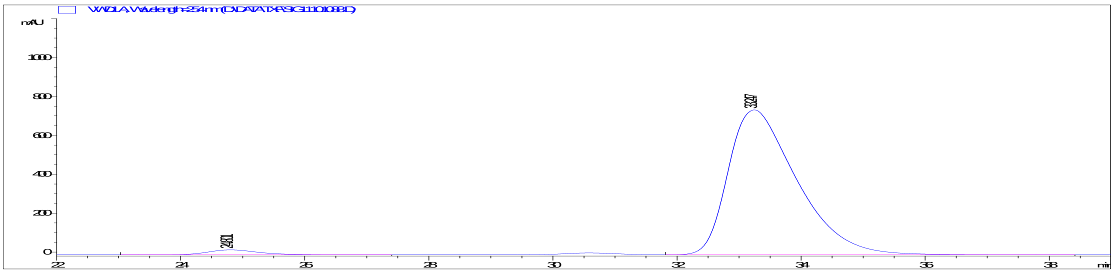

Peak #	Ret.Time (min)	Area mAU *S	Height (mAU)	Width (min)	Symmetry Factor	Area (%)
1	24.801	1515.9	25.5	0.892	0.756	2.577
2	33.247	57306.3	744.6	1.157	0.574	97.423

((2*R*,3*S*)-3-(Difluoro(phenylsulfonyl)methyl)-1-(4-methoxyphenyl)aziridin-2-yl)(naphthalen-2-yl)methanone (4h)

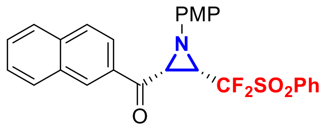

The *cis*-CF_2-aziridine **4h** was obtained as white solid after neutral alumina column chromatography (petroleum ether/dichloromethane = 2:1), 70 mg, 47% yield, 50% ee; M.p. 163.6-164.5 °C; R_f = 0.2 (dichloromethane/ petroleum ether = 3:1); **4h** with 50% ee (70 mg) was dissolved in an appropriate amount of isopropanol (0.2 mL/mg) with ultrasound for 3 minutes, followed by filtration, and the obtained solution was concentrated to give **4h** with 99% ee; Repeating the above operation led to 99% ee analyzed by HPLC; The obtained solution was combined and concentrated to give **4h** (22% yield, 99% ee).

^{1}H NMR (400 MHz, $CDCl_3$) δ 8.71 (s, 1H), 8.12 (dd, *J* = 8.6, 1.4 Hz, 1H), 7.99 – 7.82 (m, 5H), 7.67 (t, *J* = 7.5 Hz, 1H), 7.60 (t, *J* = 7.4 Hz, 1H), 7.57 – 7.48 (m, 3H), 7.31 – 7.21 (m, 2H), 6.88 (d, *J* = 8.8 Hz, 2H), 3.83 – 3.76 (m, 4H), 3.65 (dt, *J* = 15.8, 6.3 Hz, 1H). **^{13}C NMR** (101 MHz, $CDCl_3$) δ 190.5, 156.8, 143.9, 136.1, 135.8, 132.8, 132.5, 132.0, 131.3, 130.9, 130.0, 129.5, 129.1, 128.7, 127.9, 127.1, 124.1, 121.3, 120.4 (dd, J_{C-F} = 291.5, 283.9 Hz), 114.8, 55.7, 45.7, 42.3 (dd, J_{C-F} = 30.0, 20.5 Hz). **^{19}F NMR** (377 MHz, $CDCl_3$) δ -102.28 – -103.67 (m), -106.15 (ddd, *J* = 237.3, 15.8, 2.9 Hz). **HRMS** (ESI) m/z calcd for $C_{27}H_{22}NO_4F_2S$ $[M+H]^+$: 494.1238, found: 494.1235; $[\alpha]_D^{20}$ = 107.4 (c 1.0, CH_2Cl_2), 99% ee; **HPLC** (column, Daicel Chirapak IC, n-Hexane/i-PrOH = 70/30, flow rate 1.0 mL/min, 20 °C detection UV 254 nm) t_R of major isomer 48.4 min, t_R of minor isomer 32.4 min.

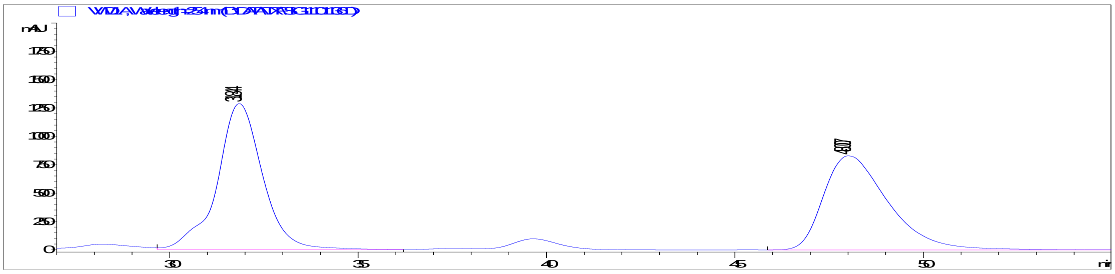

Peak #	Ret.Time (min)	Area mAU *S	Height (mAU)	Width (min)	Symmetry Factor	Area (%)
1	31.844	107260.8	1285.3	1.2532	0.932	51.877
2	48.007	99500.1	830.4	1.8288	0.582	48.123

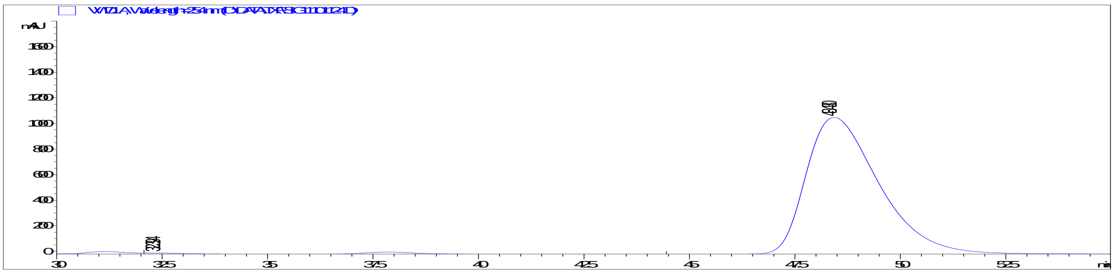

Peak #	Ret.Time (min)	Area mAU *S	Height (mAU)	Width (min)	Symmetry Factor	Area (%)
1	32.394	629.7	9.5	0.9579	0.375	0.487
2	48.43	128714.2	1068.8	1.8593	0.586	99.513

2.3 Preparation of compound 5a[6]

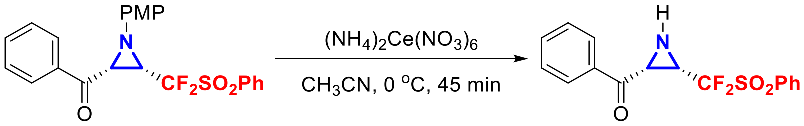

A solution of **4a** (44.3 mg, 0.1 mmol, >99.5% ee) in CH_3CN (2 mL) in a Schlenk tube (25 mL) under an argon atmosphere was placed in an ice water bath and $(NH_4)_2Ce(NO_3)_6$ (185 mg, 0.3 mmol) in H_2O (1.4 mL) was added dropwise. After stirring at that temperature for 45 minutes, the reaction mixture was quenched with saturated aq Na_2SO_3, and extracted three times with CH_2Cl_2. The combined organic layer was washed with water and brine, and then dried over anhydrous Na_2SO_4, filtered and evaporated under vacuum. The obtained solid was purified by silica gel column chromatography (eluting with petroleum ether/ethyl acetate) to give ((2*R*,3*S*)-3-(difluoro(phenylsulfonyl)methyl)aziridin-2-yl)(phenyl)methanone (**5a**) as white solid with 81% yield, >99.9% ee. M.p. 165.8-166.4 °C; R_f = 0.2 (petroleum ether/ethyl acetate = 5:1).

((2*R*,3*S*)-3-(Difluoro(phenylsulfonyl)methyl)aziridin-2-yl)(phenyl)methanone (5a)

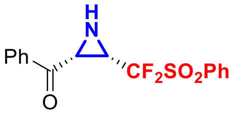

^{1}H NMR (400 MHz, $CDCl_3$) δ 8.07 (d, *J* = 7.4 Hz, 2H), 7.91 (d, *J* = 7.8 Hz, 2H), 7.73 (t, *J* = 7.5 Hz, 1H), 7.56 (tt, *J* = 23.4, 7.4 Hz, 5H), 3.64 (d, *J* = 5.1 Hz, 1H), 3.33 (s, 1H), 2.37 (s, 1H). **^{13}C NMR** (101 MHz, $CDCl_3$) δ 191.3, 135.9, 135.5, 134.2, 131.9, 131.0, 129.5, 128.9, 128.9, 120.8 (t, J_{C-F} = 289.0 Hz), 39.0, 36.7 (t, J_{C-F} = 25.2 Hz). **^{19}F NMR** (377 MHz, $CDCl_3$) δ -103.42 (dd, *J* = 235.3, 11.9 Hz), -104.66 (d, *J* = 234.5 Hz). **HRMS** (ESI) m/z calcd for $C_{16}H_{14}NO_3F_2S$ $[M+H]^+$: 338.0662, found: 338.0670; $[\alpha]_D^{20}$ = 116.4 (c 1.0, CH_2Cl_2), >99.9% ee; **HPLC** (column, Daicel Chirapak IC, n-Hexane/i-

PrOH = 70/30, flow rate 1.0 mL/min, 20 °C detection UV 254 nm) t_R of major isomer 20.5 min, t_R of minor isomer 36.3 min.

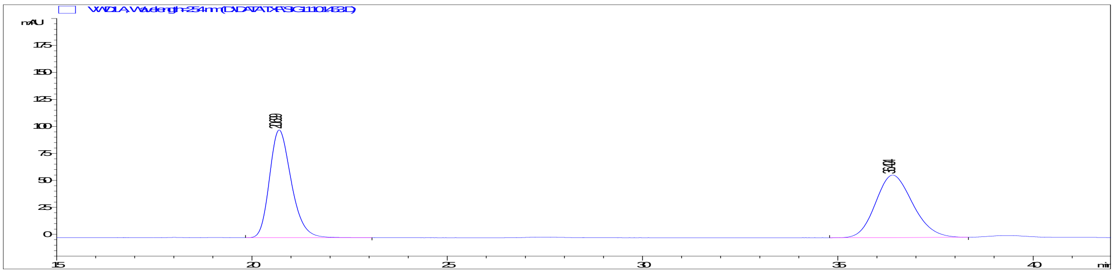

Peak #	Ret.Time (min)	Area mAU *S	Height (mAU)	Width (min)	Symmetry Factor	Area (%)
1	20.699	3870.3	99.8	0.5922	0.748	50.352
2	36.404	3816.2	57.8	1.0235	0.818	49.648

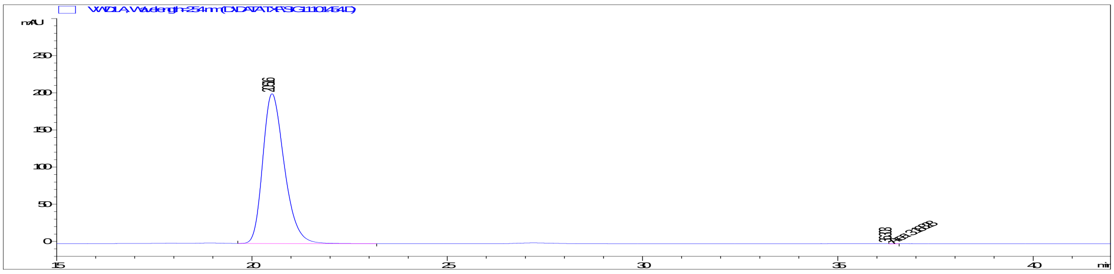

Peak #	Ret.Time (min)	Area mAU *S	Height (mAU)	Width (min)	Symmetry Factor	Area (%)
1	20.516	7726.7	201.6	0.5899	0.708	99.959
2	36.303	3.2	3.1E-1	0.1296	0	0.041

2.4 Preparation of compound 5b[7]

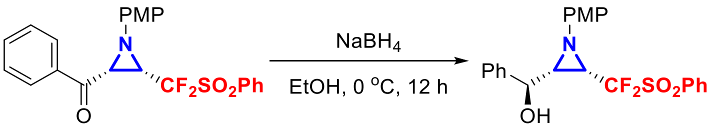

To a solution of **4a** (44.3 mg, 0.1 mmol, >99.5% ee) in EtOH (2 mL) in a round-bottom flask (10 mL) at 0 °C was added $NaBH_4$ (6 mg, 0.15 mmol) in small amounts. After being stirred at that temperature for 12 h, the reaction mixture was quenched by addition of H_2O and acidified with 4 M aq HCl, and extracted three times with CH_2Cl_2. The combined organic layer was washed with water and brine, and then dried over anhydrous Na_2SO_4, filtered and evaporated under vacuum. The residue was purified by silica gel column chromatography (eluting with petroleum ether/ethyl acetate) to give ((2*R*,3*S*)-3-(difluoro(phenylsulfonyl)methyl)aziridin-2-yl)(phenyl)methanone (**5b**) as

white solid with 95% yield, >99.5% ee. M.p. 39.0-40.0 °C; R_f = 0.6 (petroleum ether/ethyl acetate = 5:1).

(*S*)-((2*R*,3*S*)-3-(Difluoro(phenylsulfonyl)methyl)-1-(4-methoxyphenyl)aziridin-2-yl)(phenyl)methanol (5b)

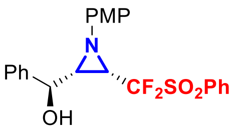

^{1}H NMR (400 MHz, $CDCl_3$) δ 8.10 (d, *J* = 7.6 Hz, 2H), 7.84 (t, *J* = 7.5 Hz, 1H), 7.70 (t, *J* = 7.8 Hz, 2H), 7.61 (d, *J* = 7.1 Hz, 2H), 7.45 (t, *J* = 7.3 Hz, 2H), 7.42 – 7.36 (m, 1H), 6.65 – 6.57 (m, 2H), 6.39 (d, *J* = 8.9 Hz, 2H), 4.83 (d, *J* = 9.0 Hz, 1H), 3.73 – 3.61 (m, 4H), 3.02 (dt, *J* = 18.8, 6.0 Hz, 1H), 2.73 – 2.66 (m, 1H). **^{13}C NMR** (101 MHz, $CDCl_3$) δ 156.1, 144.7, 141.5, 136.2, 131.5, 131.1, 129.8, 128.8, 128.3, 126.4, 122.0 (t, $J_{C\text{-}F}$ = 287.6 Hz), 120.4, 114.4, 71.5 (d, *J* = 5.8 Hz), 55.5, 51.4, 40.6 (dd, $J_{C\text{-}F}$ = 25.5, 19.2 Hz). **^{19}F NMR** (377 MHz, $CDCl_3$) δ -95.94 (dd, *J* = 236.0, 4.1 Hz), -108.40 (dd, *J* = 236.0, 18.9 Hz). **HRMS** (ESI) m/z calcd for $C_{23}H_{22}NO_4F_2S$ $[M+H]^+$: 446.1238, found: 446.1233; $[\alpha]_D^{20}$ = -83.0 (c 1.0, CH_2Cl_2), >99% ee; **HPLC** (column, Daicel Chirapak IC, n-Hexane/i-PrOH = 80/20, flow rate 1.0 mL/min, 20 °C detection UV 254 nm) t_R of major isomer 11.4 min, t_R of minor isomer 8.5 min. The stereochemistry of this compound was determined based on the ^{1}H-NMR, ^{19}F-NMR, and 2D-NOE analysis. In the 2D-NOE spectrum, the correlation between HO (4.83) and HC-N (2.7) was observed, whereas no correlation between HC-O (3.7) and HC-CF_2 (3.02) was found. See the spectrum part for details.

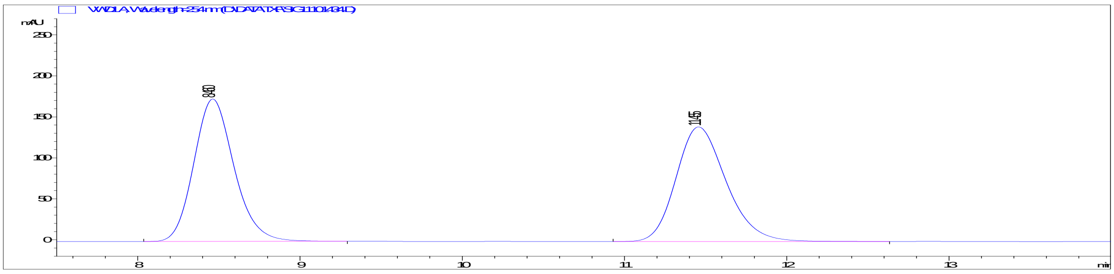

Peak #	Ret.Time (min)	Area mAU *S	Height (mAU)	Width (min)	Symmetry Factor	Area (%)
1	8.46	2994	173.8	0.2651	0.833	49.926
2	11.455	3003	139.9	0.3291	0.777	50.074

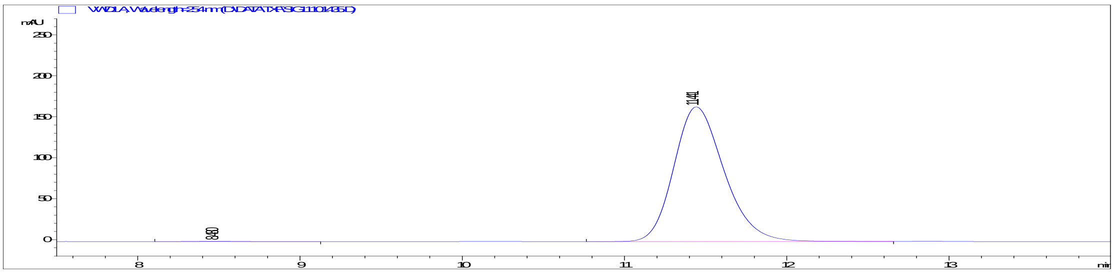

Peak #	Ret.Time (min)	Area mAU *S	Height (mAU)	Width (min)	Symmetry Factor	Area (%)
1	8.48	5.3	2.8E-1	0.2694	0.926	0.148
2	11.441	3542.3	164.5	0.3328	0.769	99.852

2.5 Preparation of compound 5c[8]

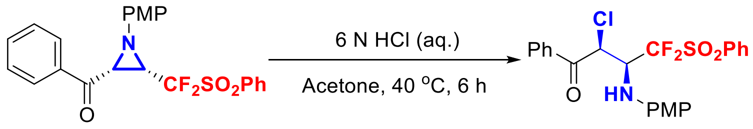

The *cis*-CF_2-aziridine **4a** (44.3 mg, 0.1 mmol, >99.5% ee) was dissolved in acetone (4 mL) in a two-necked round-bottom flask. To this solution was added 6 N aq HCl (2 mL) dropwise at room temperature. The flask was then equipped with an air condenser and an argon balloon at the top of the condenser through a rubber septum. The solution was stirred at 40 °C for 6 hours until the consumption of **4a** was completed (monitored by TLC). The solution was then cooled to room temperature and quenched with saturated aq $NaHCO_3$ until no bubbles were generated, and extracted three times with CH_2Cl_2. The combined organic layer was washed with water and brine, and then dried over anhydrous Na_2SO_4, filtered and evaporated under vacuum. The obtained solid was dissolved in CH_2Cl_2 (1 mL), and performed two-phase recrystallization with 45 mL of petroleum ether at room temperature. The (2*S*,3*R*)-2-chloro-4,4-difluoro-3-((4-methoxyphenyl)amino)-1-phenyl-4-(phenylsulfonyl)butan-1-one (**5c**) was collected as white solid with 89% yield, >99.9% ee. M.p. 140.3-141.2 °C; R_f = 0.4 (petroleum ether/ethyl acetate = 5/1).

(2*S*,3*R*)-2-Chloro-4,4-difluoro-3-((4-methoxyphenyl)amino)-1-phenyl-4-(phenylsulfonyl)butan-1-one (5c)

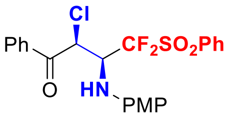

^{1}H NMR (400 MHz, DMSO-d_6) δ 7.92 (d, *J* = 7.5 Hz, 2H), 7.85 (t, *J* = 8.5 Hz, 3H), 7.73 (t, *J* = 7.4 Hz, 1H), 7.65 (t, *J* = 7.8 Hz, 2H), 7.59 (t, *J* = 7.7 Hz, 2H), 6.70 (d, *J* = 8.9 Hz, 2H), 6.58 (d, *J* = 8.9 Hz, 2H), 6.12 (d, *J* = 5.8 Hz, 1H), 5.75 (d, *J* = 11.2 Hz, 1H), 5.29 (ddt, *J* = 21.3, 10.8, 5.2 Hz, 1H), 3.66 (s, 3H). **^{13}C NMR** (101 MHz, DMSO-d_6) δ 191.1, 152.1, 139.9, 136.0, 134.4, 133.5, 132.7, 130.0, 129.7, 129.2, 128.7, 121.6 (t, $J_{C\text{-}F}$ = 295.7 Hz), 114.9, 114.3, 58.3, 55.2, 54.5 (dd, $J_{C\text{-}F}$ = 23.6, 17.8 Hz). **^{19}F NMR** (377 MHz, DMSO-d_6) δ -101.01 (dd, *J* = 234.2, 4.7 Hz), -109.11 (dd, *J* = 234.1, 20.6 Hz). **HRMS** (ESI) m/z calcd for $C_{23}H_{21}NO_4F_2SCl$ $[M+H]^+$: 480.0848, found: 480.0849; $[\alpha]_D^{20}$ = -123.6 (c 1.0, CH_2Cl_2), >99% ee; **HPLC** (column, Daicel Chirapak IC, n-Hexane/i-PrOH = 90/10, flow rate 1.0 mL/min, 20 °C detection UV 254 nm) t_R of major isomer 20.2 min, t_R of minor isomer 26.2 min.

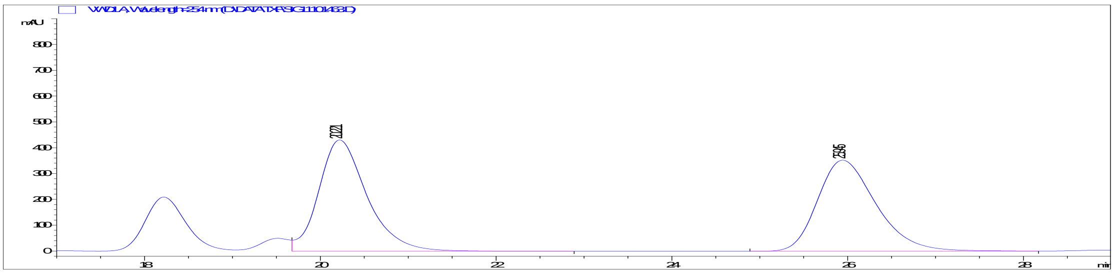

Peak #	Ret.Time (min)	Area mAU *S	Height (mAU)	Width (min)	Symmetry Factor	Area (%)
1	20.221	16447.2	430.7	0.5824	0.718	50.090
2	25.945	16388.2	352.9	0.7135	0.707	49.910

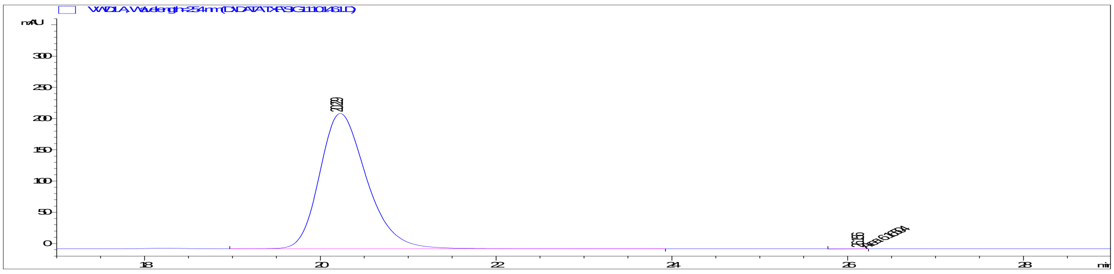

Peak #	Ret.Time (min)	Area mAU *S	Height (mAU)	Width (min)	Symmetry Factor	Area (%)
1	20.229	8066.9	216.7	0.5713	0.739	99.924
2	26.156	6.2	1.2E-1	0.6309	5.851	0.076

3 Reference

[1] Zeng, J.-L.; Zhang, F.-G.; Ma, J.-A. *Org. Lett.* **2019**, *21*, 8244.
[2] He, H.; Le, A. W. M. *Eur. J. Org. Chem.* **2010**, 4181–4184.
[3] Hashimoto, T.; Maruoka, K. *J. Am. Chem. Soc.* **2013**, *135*, 17667−17670.
[4] Ooi, T.; Uematsu, Y.; Maruoka, K. *J. Org. Chem.* **2003**, *68*, 4576.
[5] Wang, P.; Liao, S.; Tang, Y. *J. Am. Chem. Soc.* **2013**, *135*, 16849−16852.
[6] Chai, Z.; Bouillon, J.-P.; Cahard, D. *Chem. Commun.* **2012**, *48*, 9471–9473.
[7] Suzuki, T.; Mori, K.; Akiyama, T. *Org. Lett.* **2009**, *11*, 2445−2447.
[8] Gupta, A. K.; Mukherjee, M.; Wulff, W. D. *Org. Lett.* **2011**, *13*, 5866−5869.

4 NMR spectra of all the new compounds

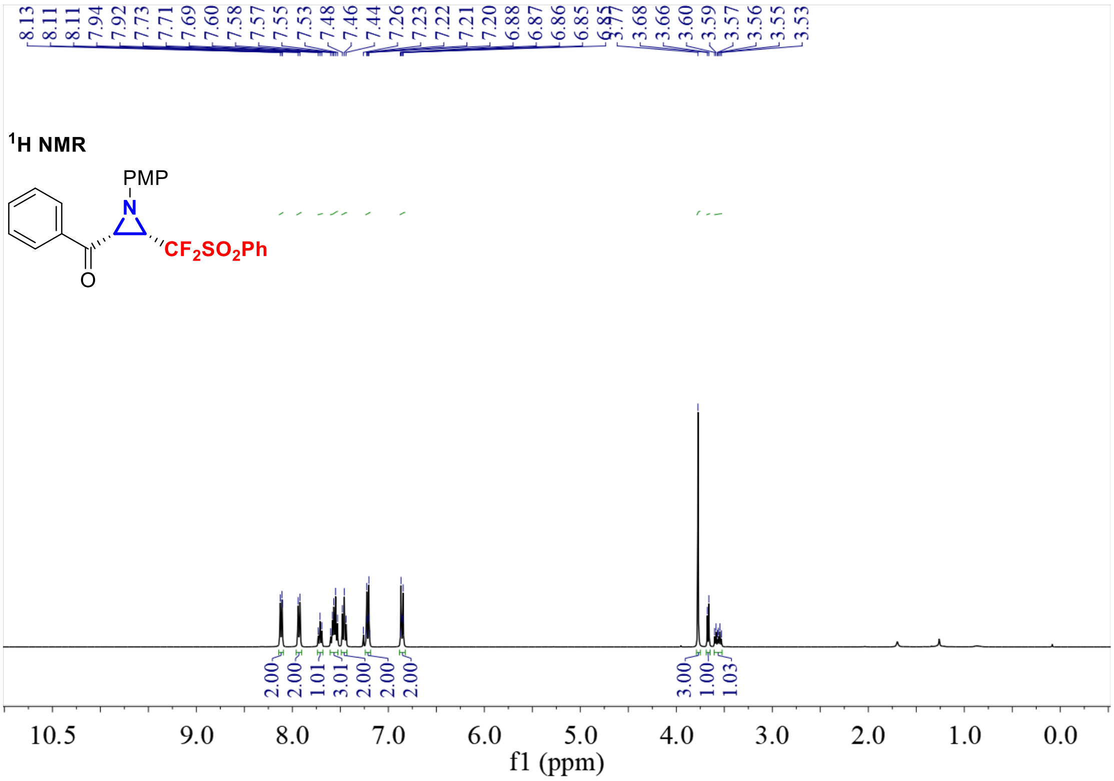

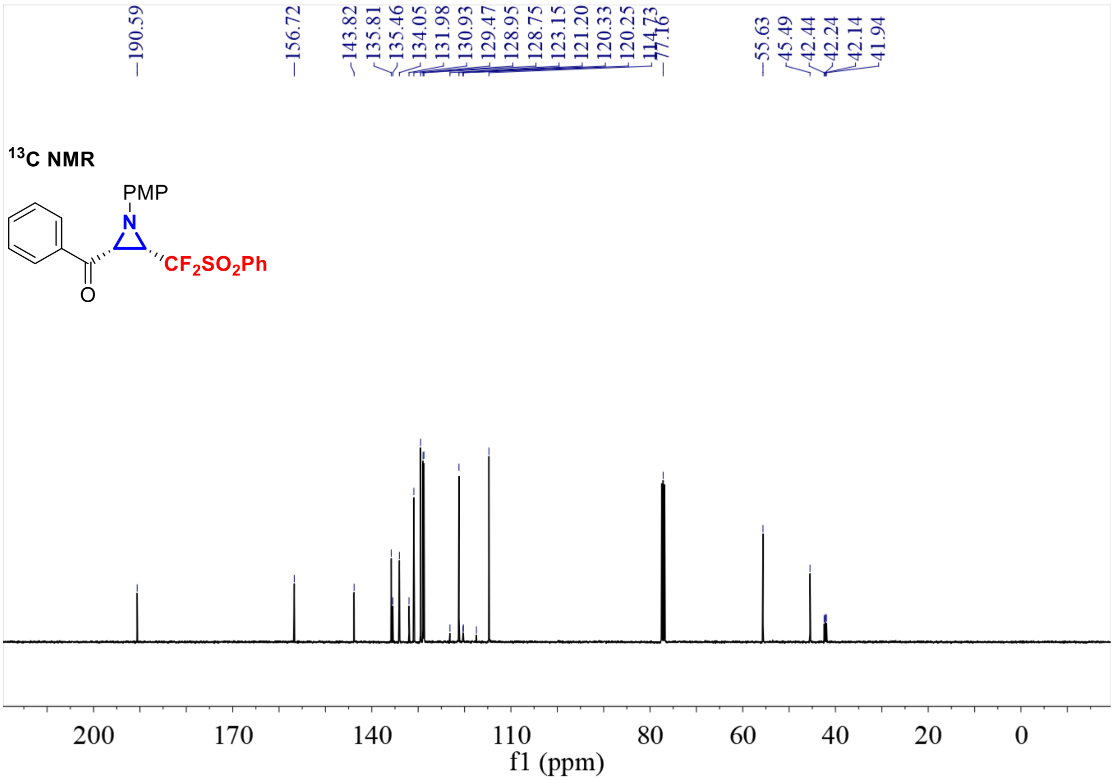

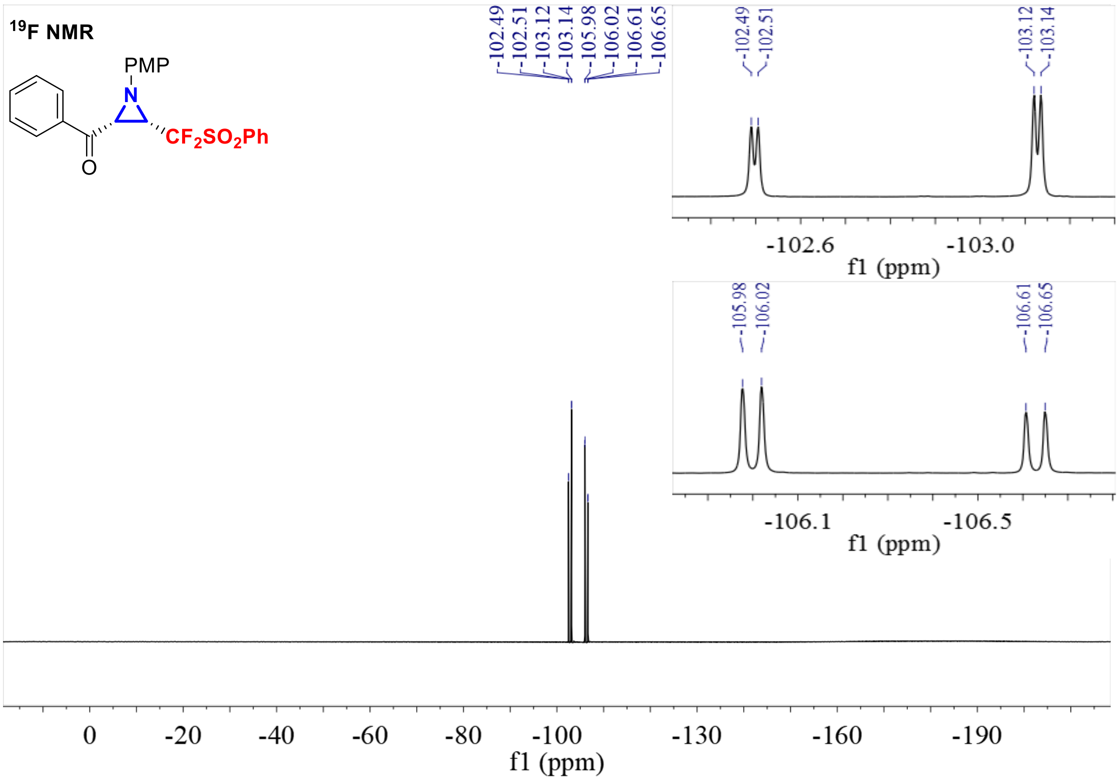

19F NMR
PMP
N
CF2SO2Ph
O
-102.49
-102.51
-103.12
-103.14
-105.98
-106.02
-106.61
-106.65
-102.6
-103.0
-106.1
-106.5
f1 (ppm)
0
-20
-40
-60
-80
-100
-130
-160
-190
f1 (ppm)

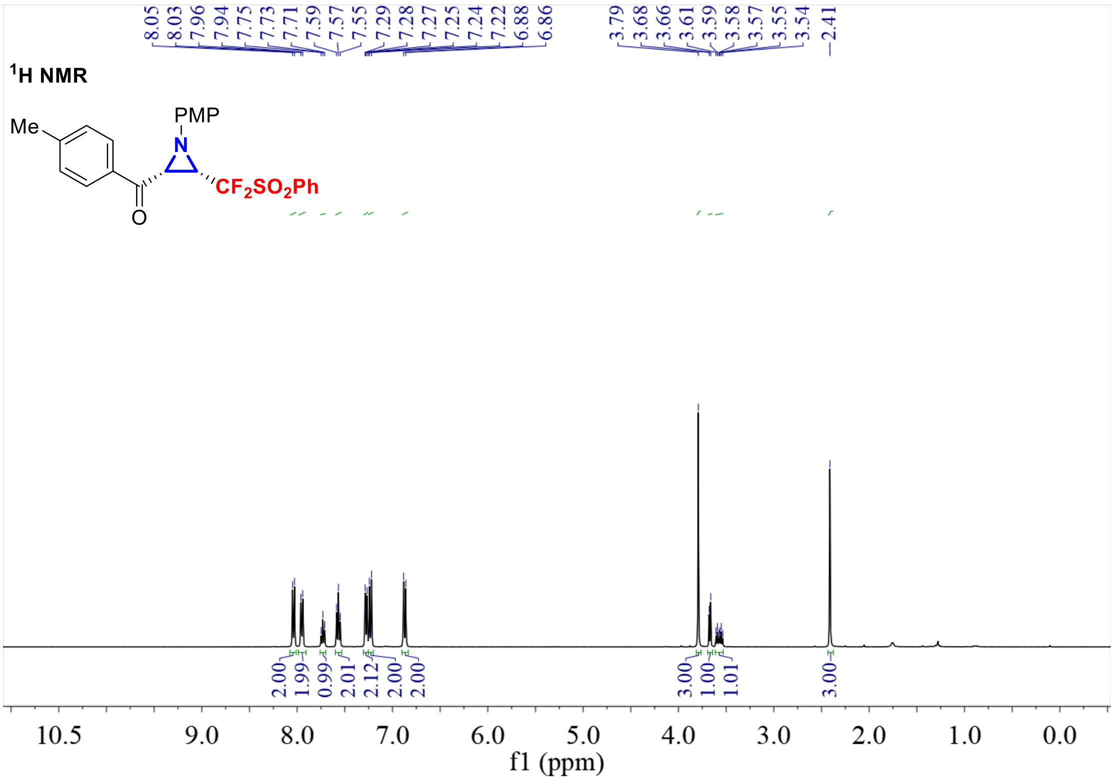

1H NMR
Me
PMP
N
CF2SO2Ph
O
8.05
8.03
7.96
7.94
7.75
7.73
7.71
7.59
7.57
7.55
7.29
7.28
7.27
7.25
7.24
7.22
6.88
6.86
3.79
3.68
3.66
3.61
3.59
3.58
3.57
3.55
3.54
2.41
2.00
1.99
0.99
2.01
2.12
2.00
2.00
3.00
1.00
1.01
3.00
10.5
9.0
8.0
7.0
6.0
5.0
4.0
3.0
2.0
1.0
0.0
f1 (ppm)

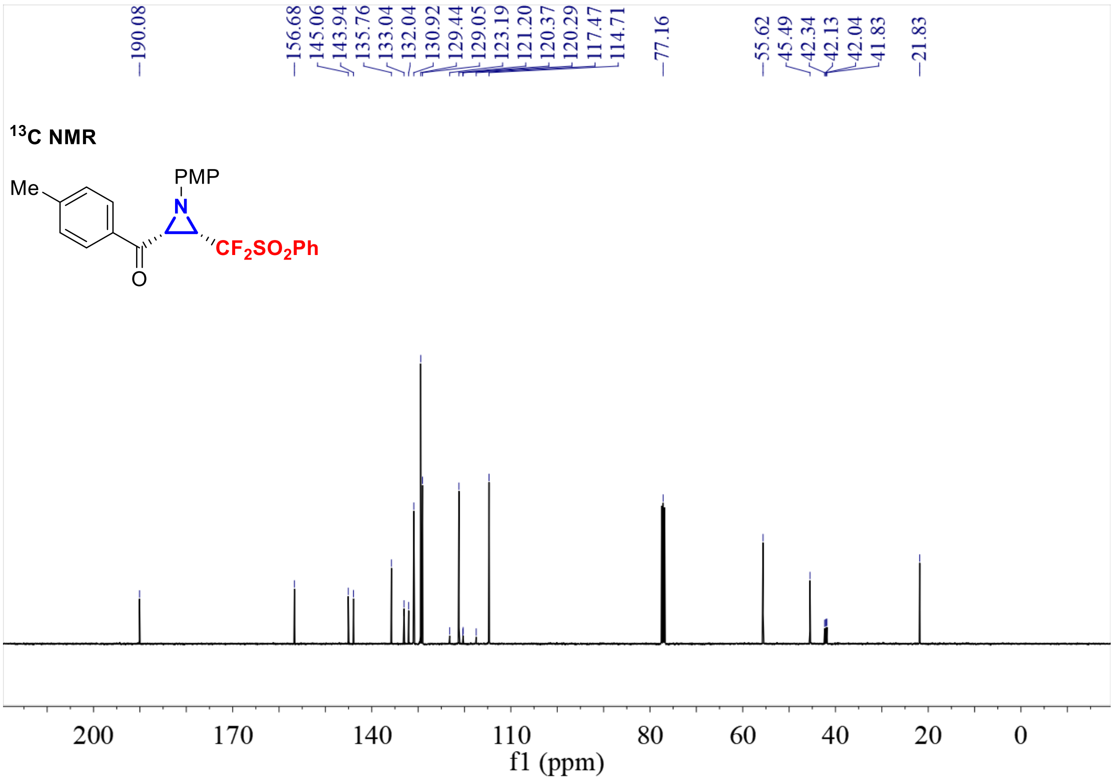
190.08
156.68
145.06
143.94
135.76
133.04
132.04
130.92
129.44
129.05
123.19
121.20
120.37
120.29
117.47
114.71
77.16
55.62
45.49
42.34
42.13
42.04
41.83
21.83
13C NMR
Me
PMP
N
O
CF2SO2Ph
200
170
140
110
80
60
40
20
0
f1 (ppm)

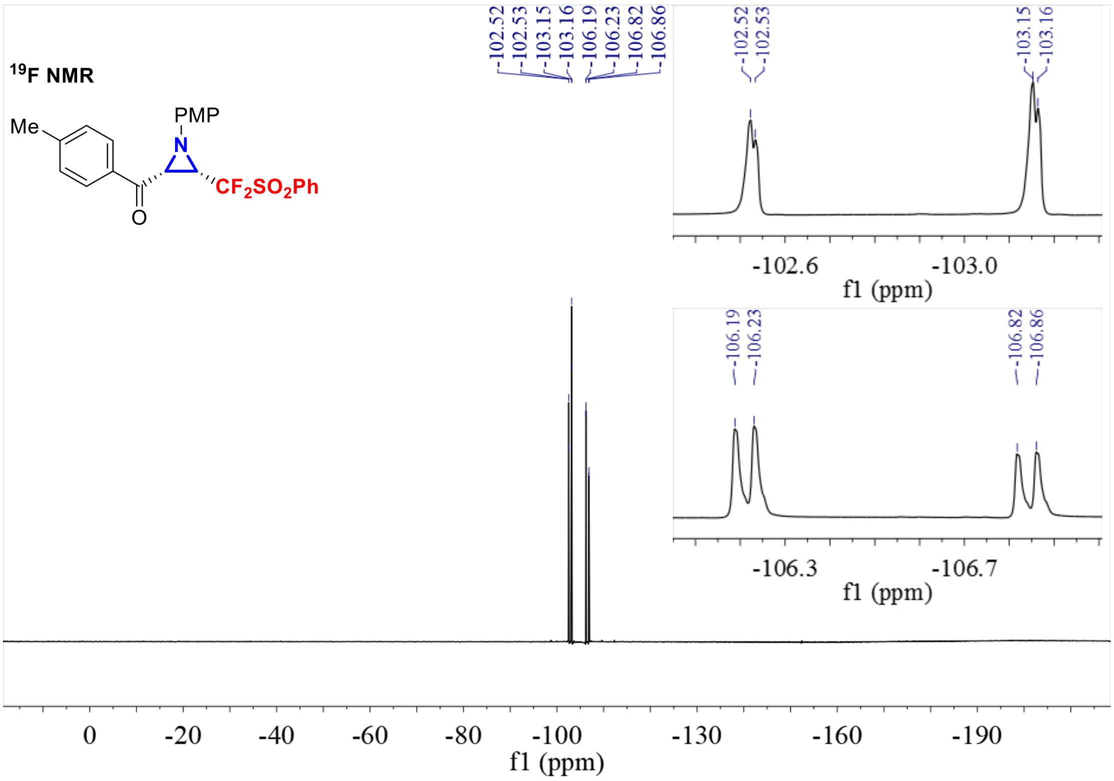
-102.52
-102.53
-103.15
-103.16
-106.19
-106.23
-106.82
-106.86
19F NMR
Me
PMP
N
O
CF2SO2Ph
-102.6
-103.0
f1 (ppm)
-106.3
-106.7
f1 (ppm)
0
-20
-40
-60
-80
-100
-130
-160
-190
f1 (ppm)

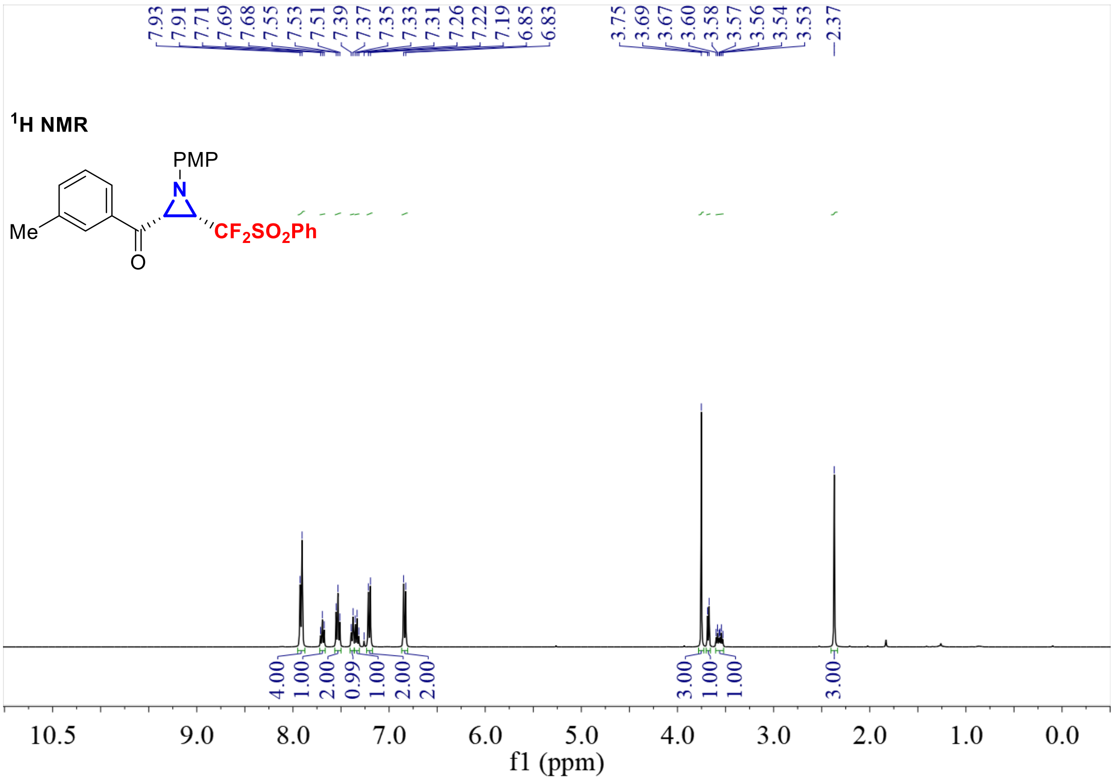

1H NMR
PMP
N
Me
O
CF2SO2Ph
f1 (ppm)

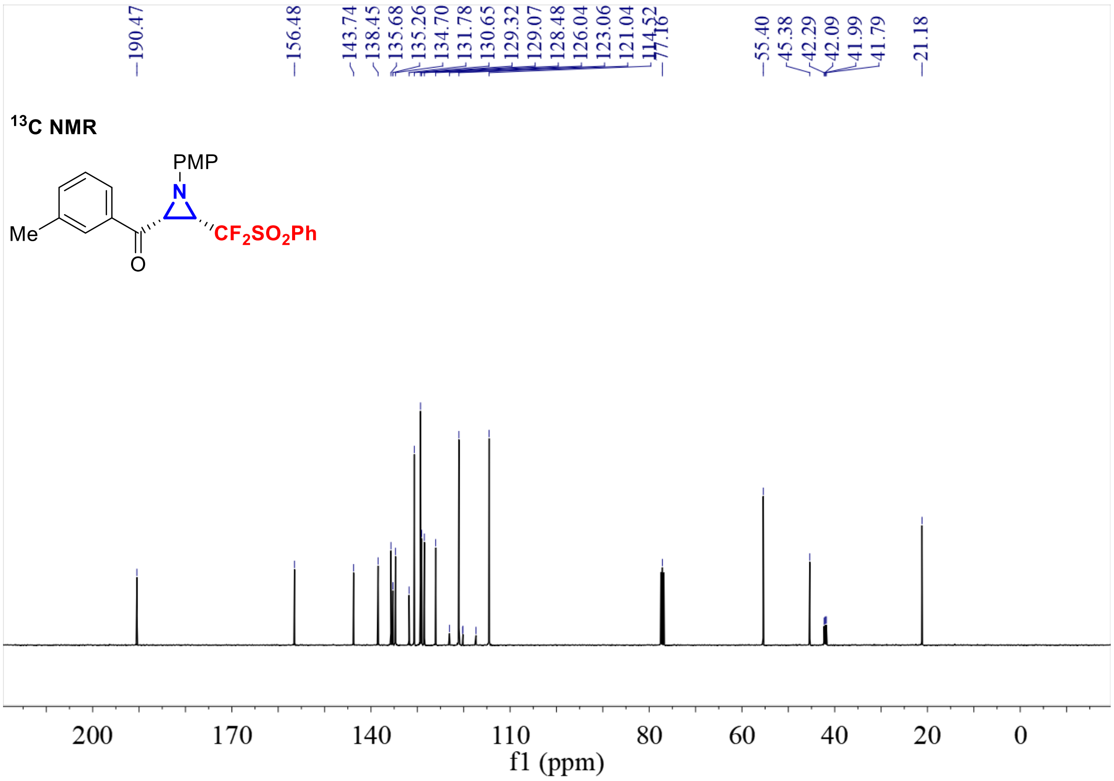

13C NMR
PMP
N
Me
O
CF2SO2Ph
f1 (ppm)

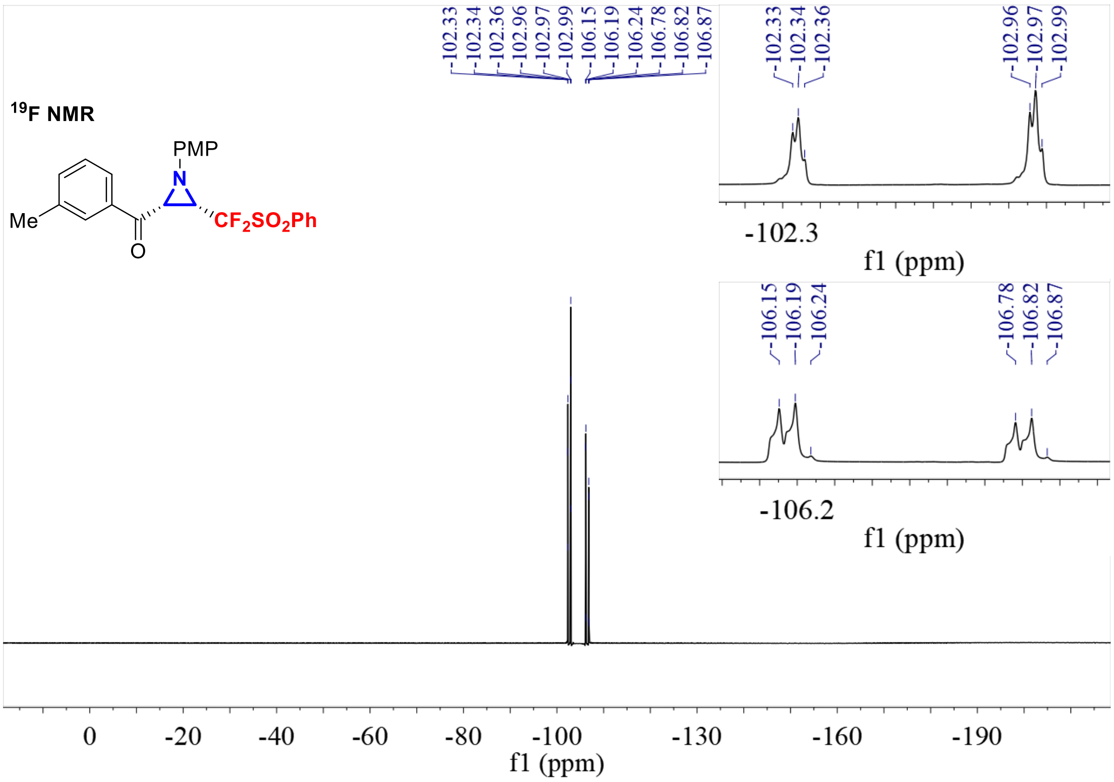

19F NMR
PMP
N
Me
O
CF2SO2Ph
-102.33
-102.34
-102.36
-102.96
-102.97
-102.99
-106.15
-106.19
-106.24
-106.78
-106.82
-106.87
-102.3
-106.2
f1 (ppm)
0
-20
-40
-60
-80
-100
-130
-160
-190

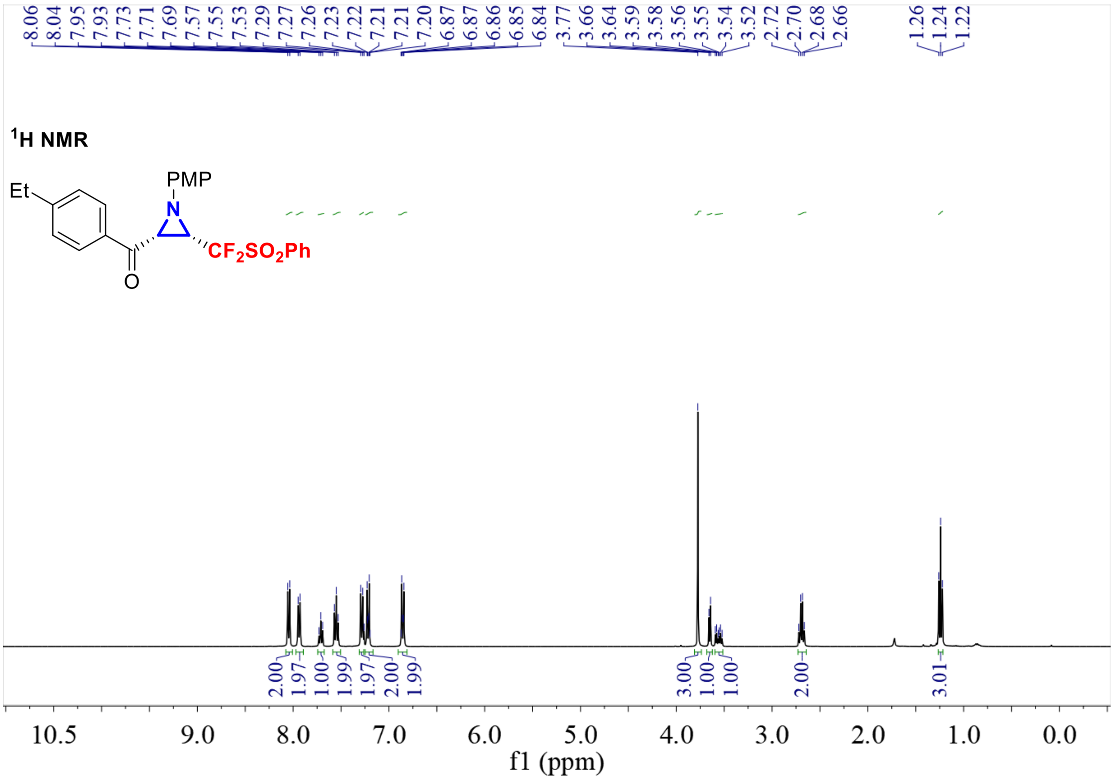

1H NMR
PMP
N
Et
O
CF2SO2Ph
8.06
8.04
7.95
7.93
7.73
7.71
7.69
7.57
7.55
7.53
7.29
7.27
7.26
7.23
7.22
7.21
7.21
7.20
6.87
6.87
6.86
6.85
6.84
3.77
3.66
3.64
3.59
3.58
3.56
3.55
3.54
3.52
2.72
2.70
2.68
2.66
1.26
1.24
1.22
2.00
1.97
1.00
1.99
1.97
2.00
1.99
3.00
1.00
1.00
2.00
3.01
10.5
9.0
8.0
7.0
6.0
5.0
4.0
3.0
2.0
1.0
0.0
f1 (ppm)

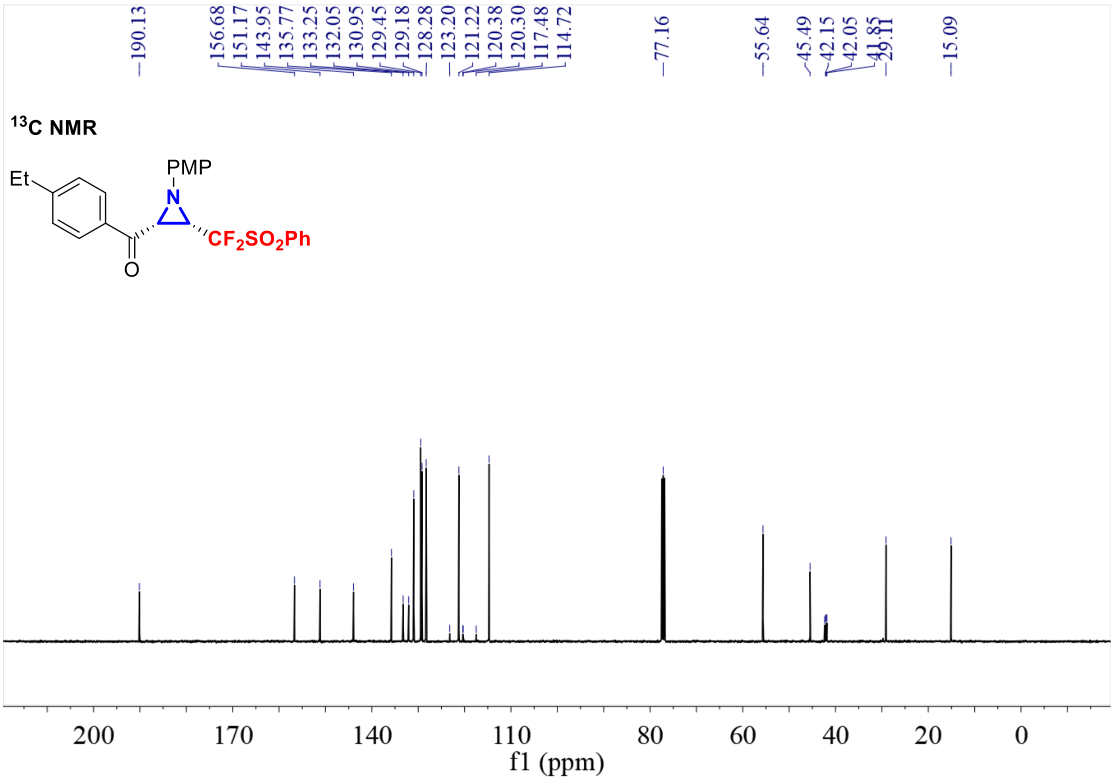

13C NMR
Et
PMP
N
CF2SO2Ph
O
190.13
156.68
151.17
143.95
135.77
133.25
132.05
130.95
129.45
129.18
128.28
123.20
121.22
120.38
120.30
117.48
114.72
77.16
55.64
45.49
42.15
42.05
41.85
29.11
15.09
f1 (ppm)

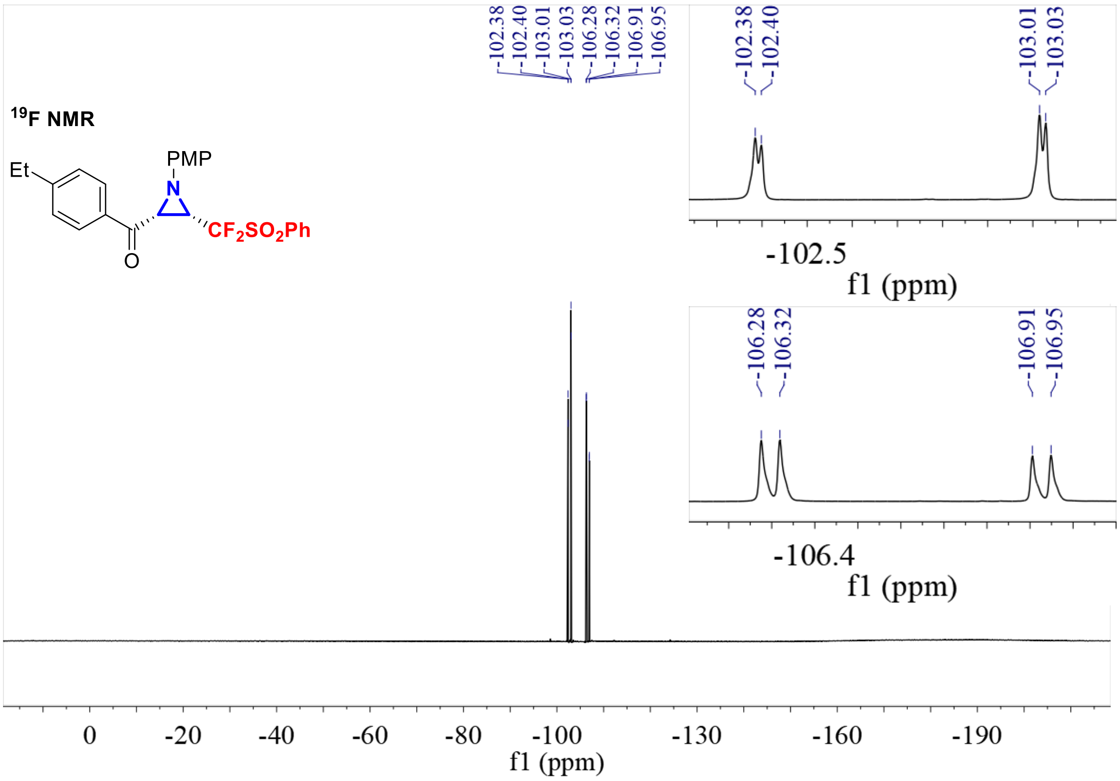

19F NMR
Et
PMP
N
CF2SO2Ph
O
-102.38
-102.40
-103.01
-103.03
-106.28
-106.32
-106.91
-106.95
-102.5
-106.4
f1 (ppm)

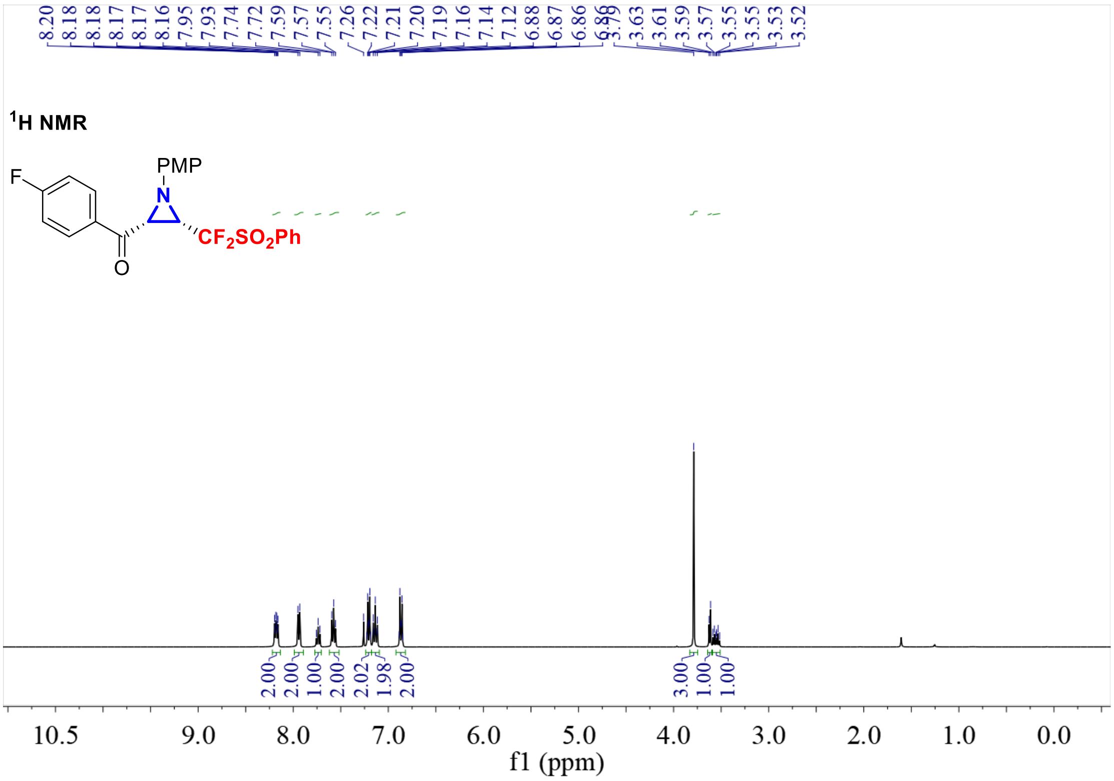

8.20
8.18
8.18
8.17
8.17
8.16
7.95
7.93
7.74
7.72
7.59
7.57
7.55
7.26
7.22
7.21
7.20
7.19
7.16
7.14
7.12
6.88
6.87
6.86
3.79
3.63
3.61
3.59
3.57
3.55
3.55
3.53
3.52
1H NMR
PMP
N
F
O
CF2SO2Ph
2.00
2.00
1.00
2.00
2.02
1.98
2.00
3.00
1.00
1.00
10.5
9.0
8.0
7.0
6.0
5.0
4.0
3.0
2.0
1.0
0.0
f1 (ppm)

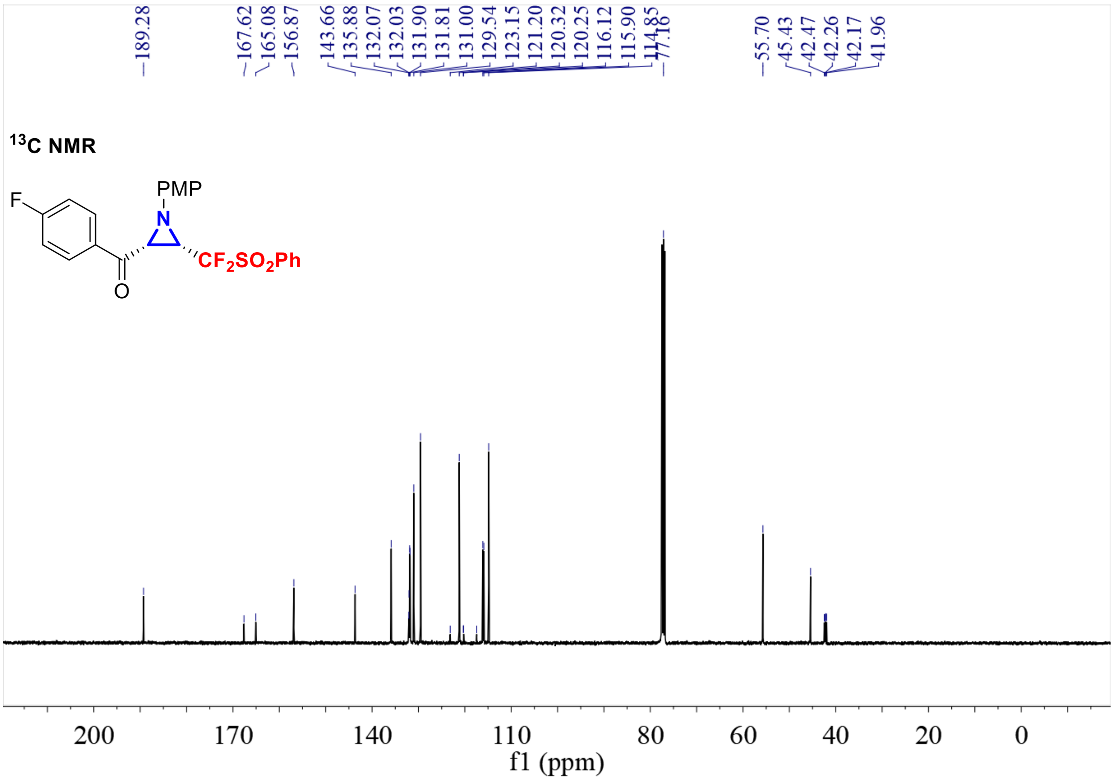

189.28
167.62
165.08
156.87
143.66
135.88
132.07
132.03
131.90
131.81
131.00
129.54
123.15
121.20
120.32
120.25
116.12
115.90
114.85
77.16
55.70
45.43
42.47
42.26
42.17
41.96
13C NMR
PMP
N
F
O
CF2SO2Ph
200
170
140
110
80
60
40
20
0
f1 (ppm)

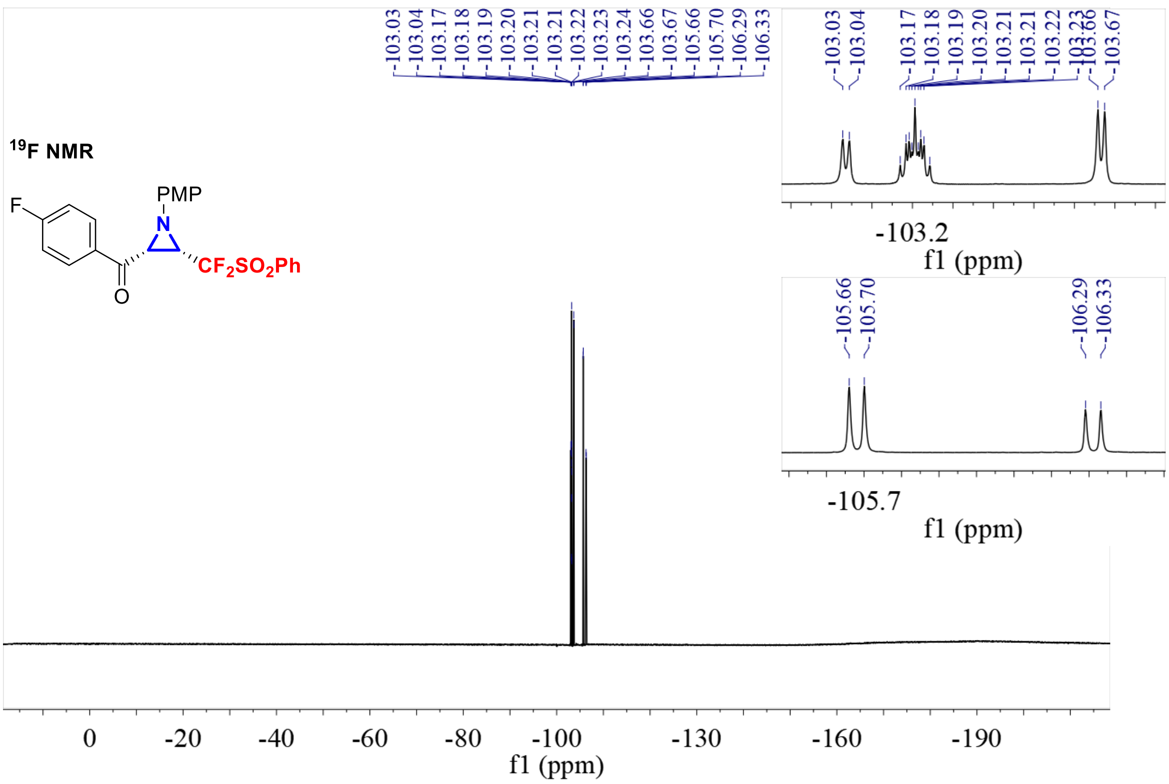
19F NMR
PMP
F
N
CF2SO2Ph
O
-103.03
-103.04
-103.17
-103.18
-103.19
-103.20
-103.21
-103.21
-103.22
-103.23
-103.24
-103.66
-103.67
-105.66
-105.70
-106.29
-106.33
-103.2
-105.7
f1 (ppm)
0
-20
-40
-60
-80
-100
-130
-160
-190
f1 (ppm)

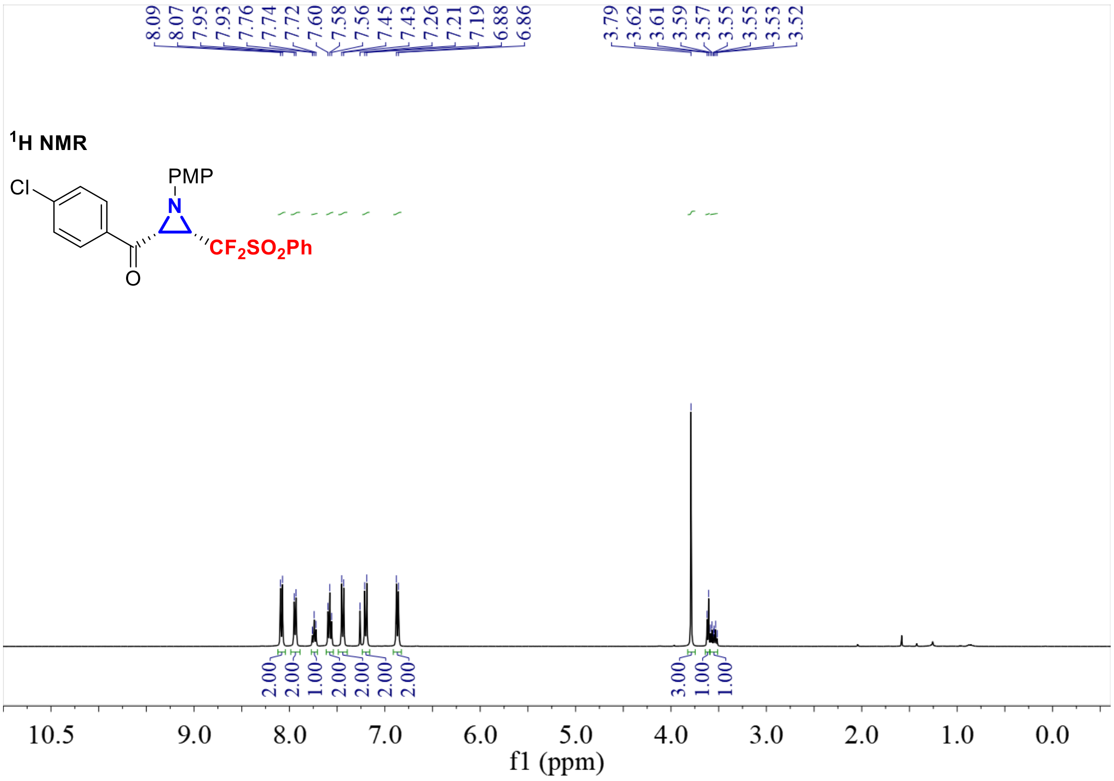
1H NMR
PMP
Cl
N
CF2SO2Ph
O
8.09
8.07
7.95
7.93
7.76
7.74
7.72
7.60
7.58
7.56
7.45
7.43
7.26
7.21
7.19
6.88
6.86
3.79
3.62
3.61
3.59
3.57
3.55
3.55
3.53
3.52
2.00
2.00
1.00
2.00
2.00
2.00
2.00
3.00
1.00
1.00
10.5
9.0
8.0
7.0
6.0
5.0
4.0
3.0
2.0
1.0
0.0
f1 (ppm)

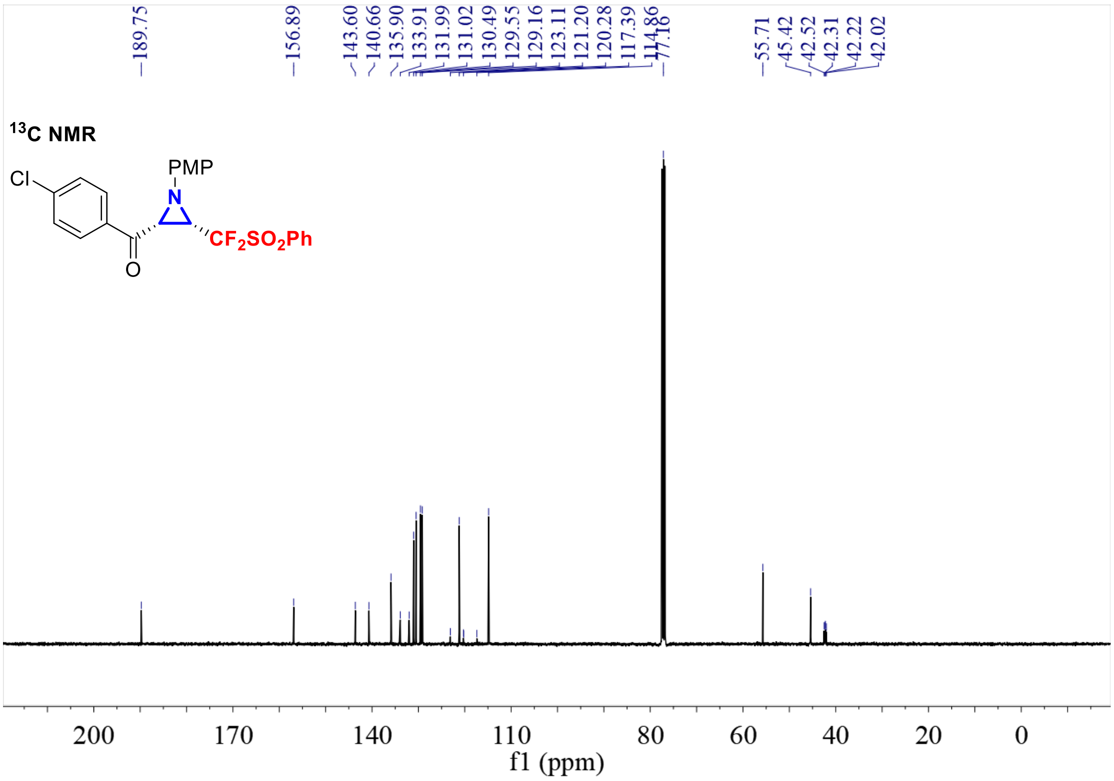
189.75
156.89
143.60
140.66
135.90
133.91
131.99
131.02
130.49
129.55
129.16
123.11
121.20
120.28
117.39
114.86
77.16
55.71
45.42
42.52
42.31
42.22
42.02
13C NMR
PMP
N
Cl
O
CF2SO2Ph
200
170
140
110
80
60
40
20
0
f1 (ppm)

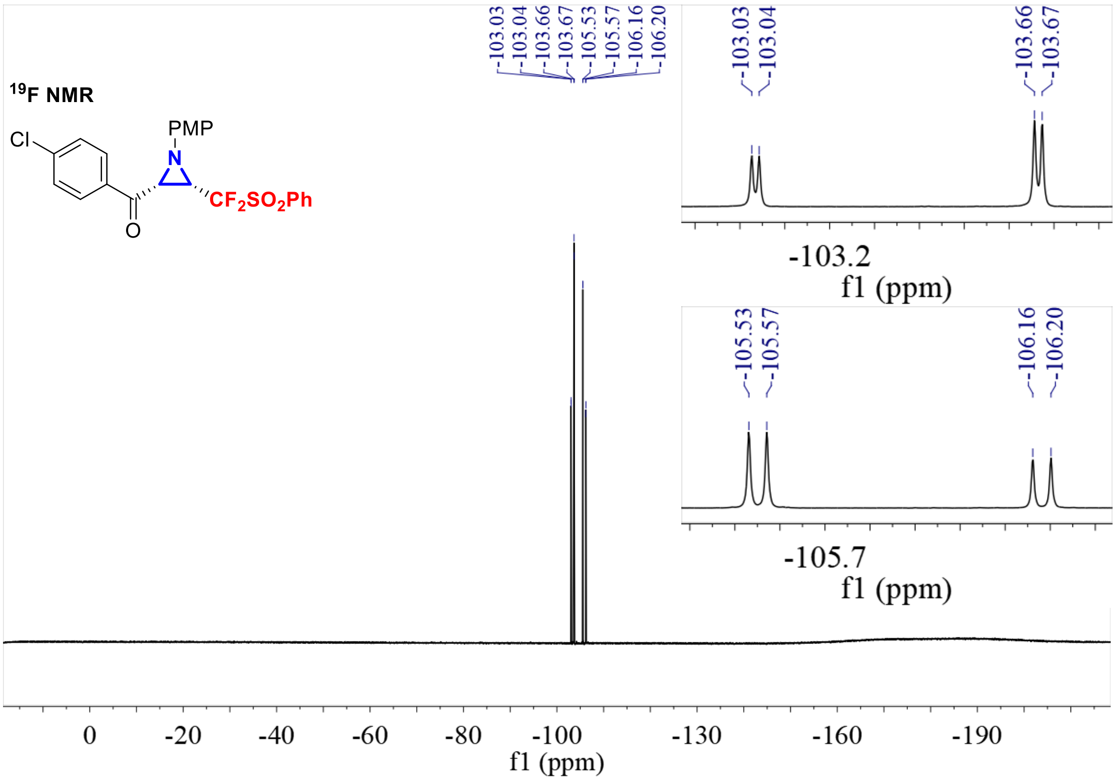
-103.03
-103.04
-103.66
-103.67
-105.53
-105.57
-106.16
-106.20
19F NMR
PMP
N
Cl
O
CF2SO2Ph
-103.2
f1 (ppm)
-105.7
f1 (ppm)
0
-20
-40
-60
-80
-100
-130
-160
-190
f1 (ppm)

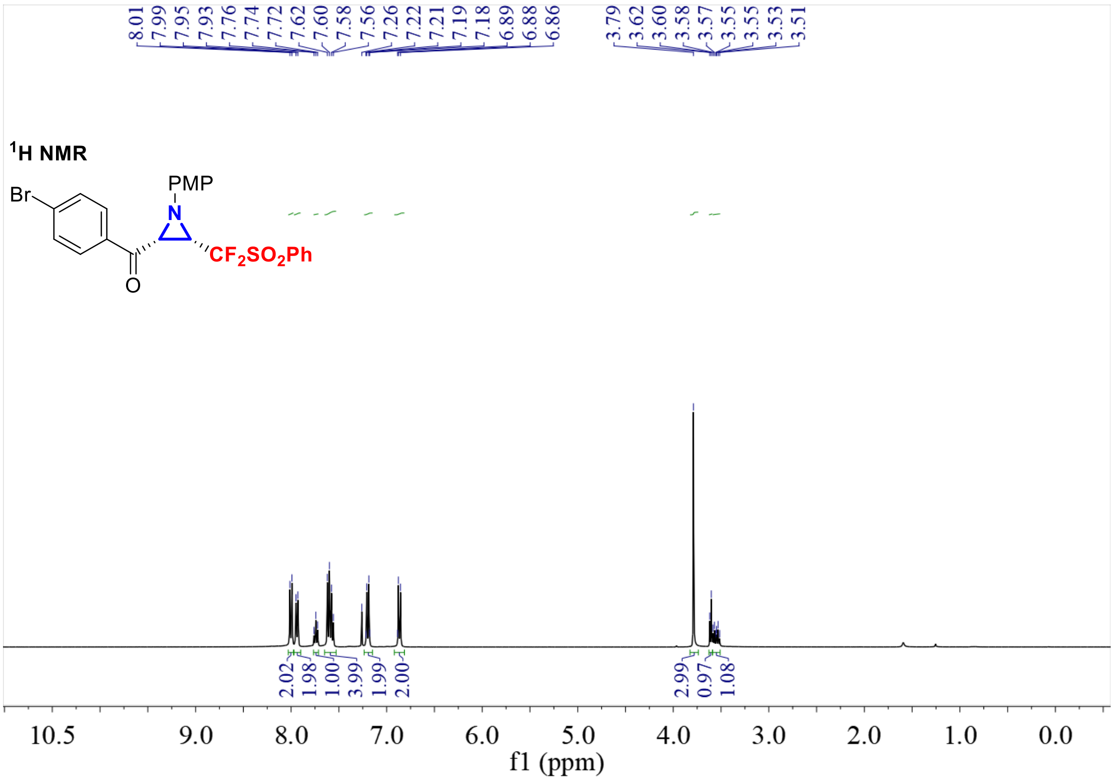

8.01
7.99
7.95
7.93
7.76
7.74
7.72
7.62
7.60
7.58
7.56
7.26
7.22
7.21
7.19
7.18
6.89
6.88
6.86
3.79
3.62
3.60
3.58
3.57
3.55
3.55
3.53
3.51
^{1}H NMR
Br
PMP
N
O
CF_2SO_2Ph
2.02
1.98
1.00
3.99
1.99
2.00
2.99
0.97
1.08
10.5
9.0
8.0
7.0
6.0
5.0
4.0
3.0
2.0
1.0
0.0
f1 (ppm)

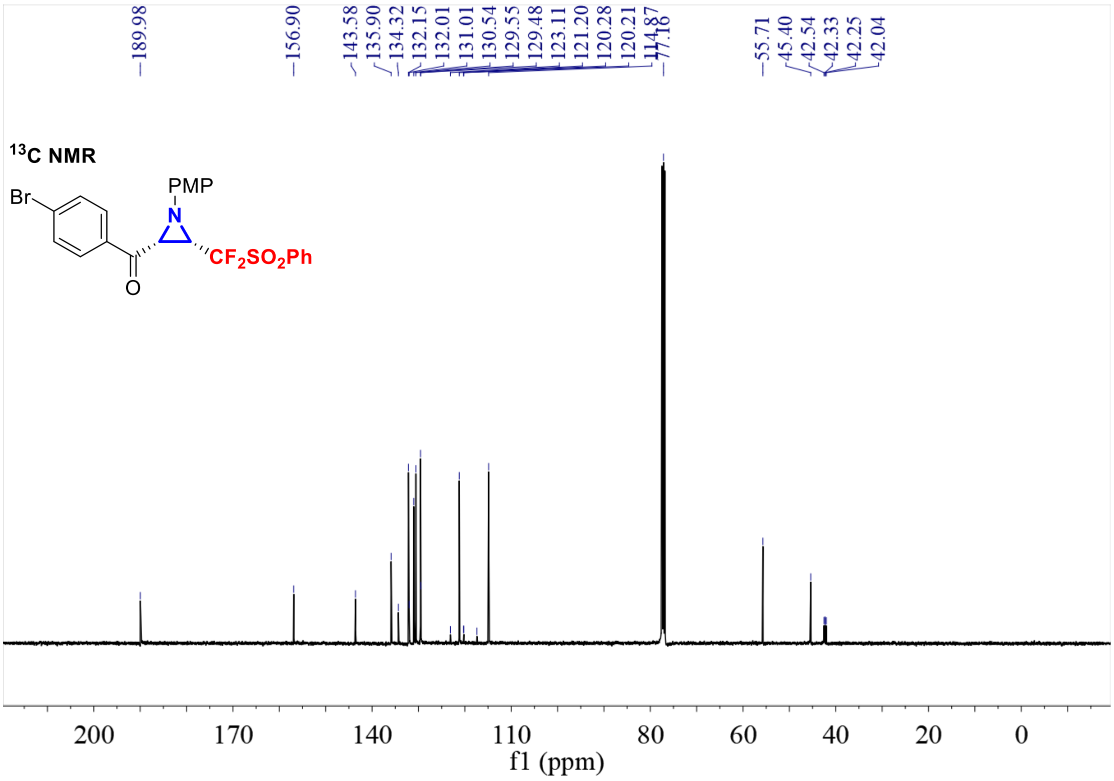

189.98
156.90
143.58
135.90
134.32
132.15
132.01
131.01
130.54
129.55
129.48
123.11
121.20
120.28
120.21
114.87
77.16
55.71
45.40
42.54
42.33
42.25
42.04
^{13}C NMR
Br
PMP
N
O
CF_2SO_2Ph
200
170
140
110
80
60
40
20
0
f1 (ppm)

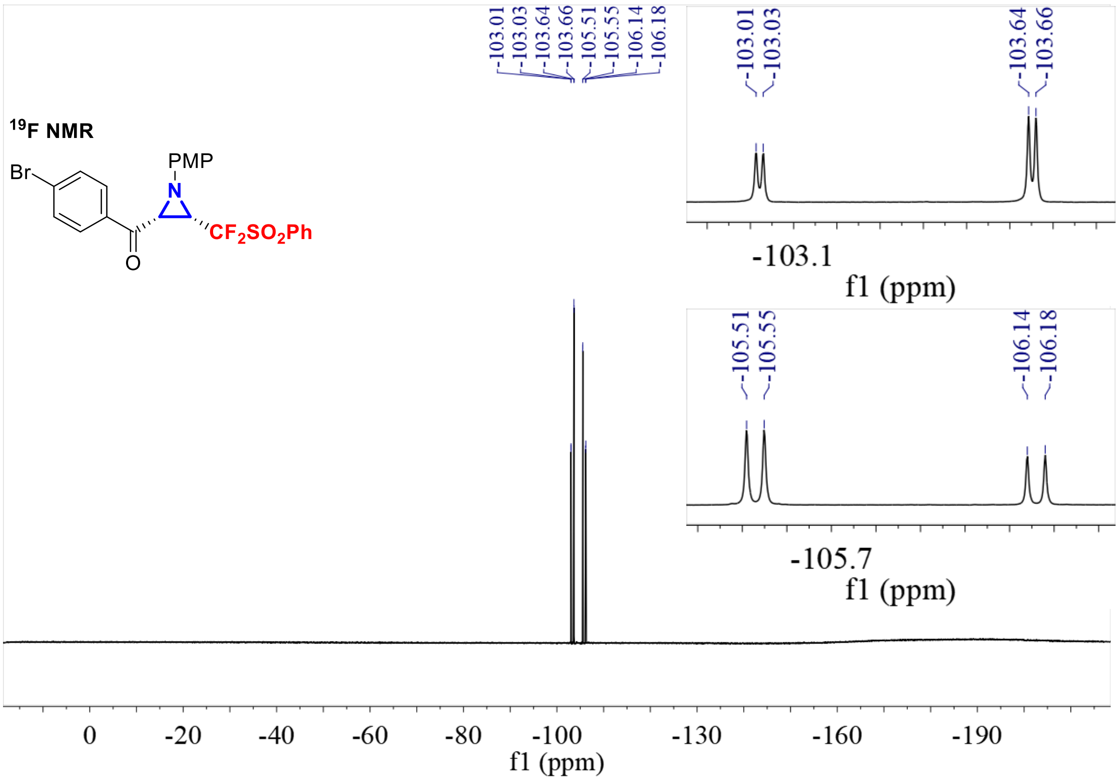

19F NMR
PMP
Br
N
CF2SO2Ph
O
-103.01
-103.03
-103.64
-103.66
-105.51
-105.55
-106.14
-106.18
-103.1
-105.7
f1 (ppm)
0
-20
-40
-60
-80
-100
-130
-160
-190

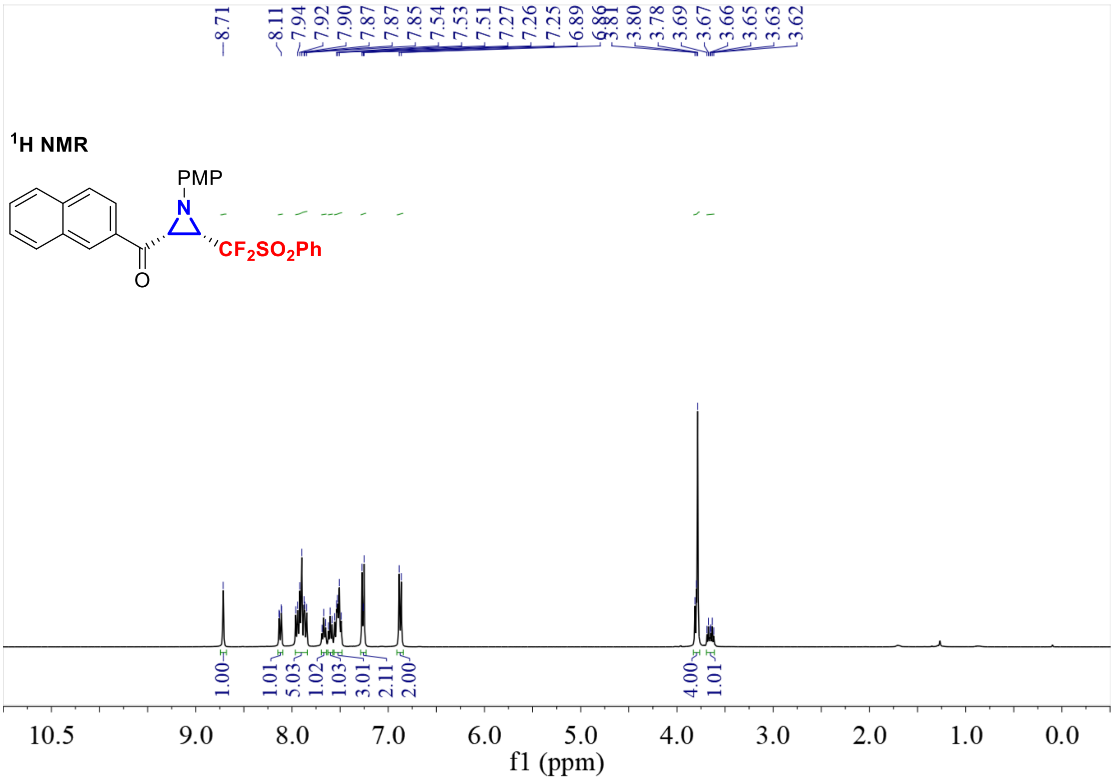

1H NMR
PMP
N
CF2SO2Ph
O
8.71
8.11
7.94
7.92
7.90
7.87
7.87
7.85
7.54
7.53
7.51
7.27
7.26
7.25
6.89
6.86
3.81
3.80
3.78
3.69
3.67
3.66
3.65
3.63
3.62
1.00
1.01
5.03
1.02
1.03
3.01
2.11
2.00
4.00
1.01
10.5
9.0
8.0
7.0
6.0
5.0
4.0
3.0
2.0
1.0
0.0
f1 (ppm)

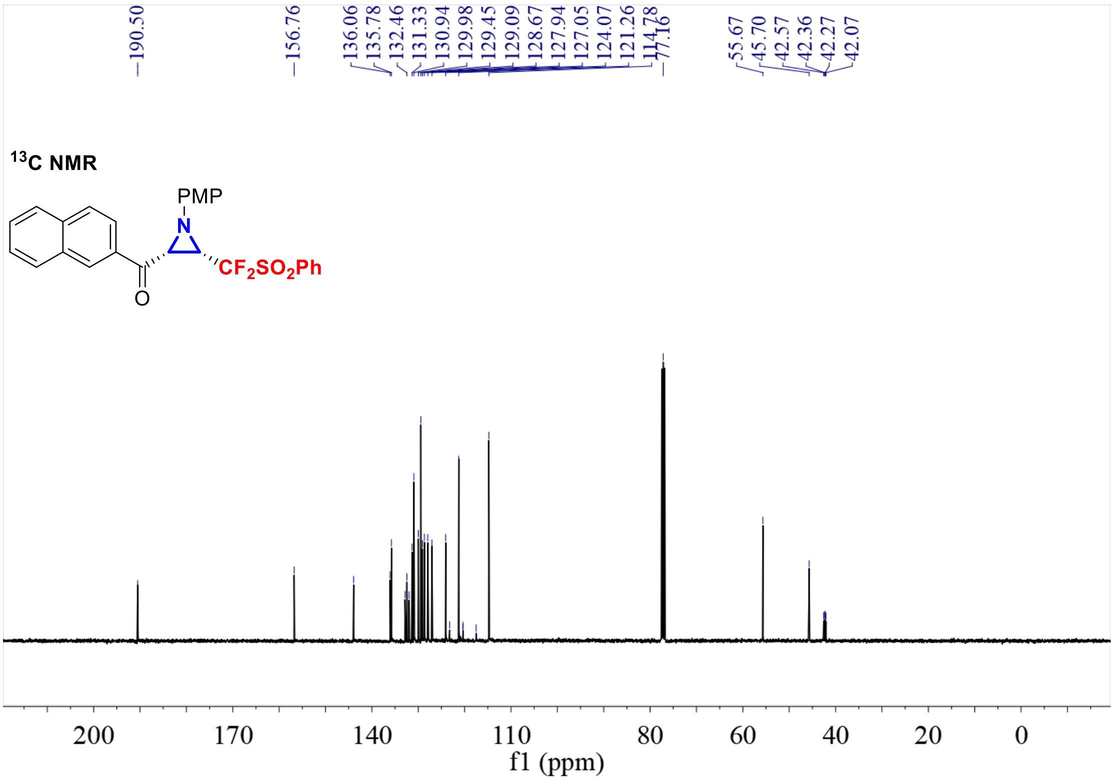

190.50
156.76
136.06
135.78
132.46
131.33
130.94
129.98
129.45
129.09
128.67
127.94
127.05
124.07
121.26
114.78
77.16
55.67
45.70
42.57
42.36
42.27
42.07
13C NMR
PMP
N
O
CF2SO2Ph
200
170
140
110
80
60
40
20
0
f1 (ppm)

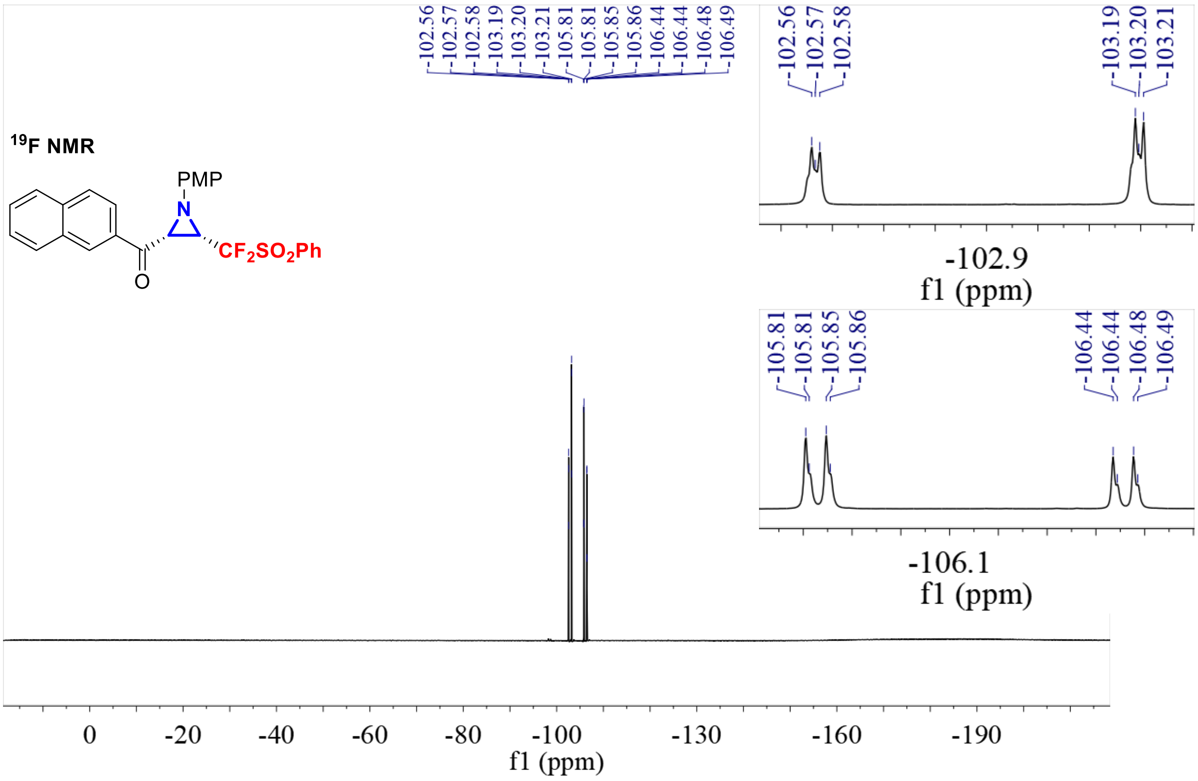

-102.56
-102.57
-102.58
-103.19
-103.20
-103.21
-105.81
-105.81
-105.85
-105.86
-106.44
-106.44
-106.48
-106.49
19F NMR
PMP
N
O
CF2SO2Ph
-102.9
f1 (ppm)
-106.1
f1 (ppm)
0
-20
-40
-60
-80
-100
-130
-160
-190
f1 (ppm)

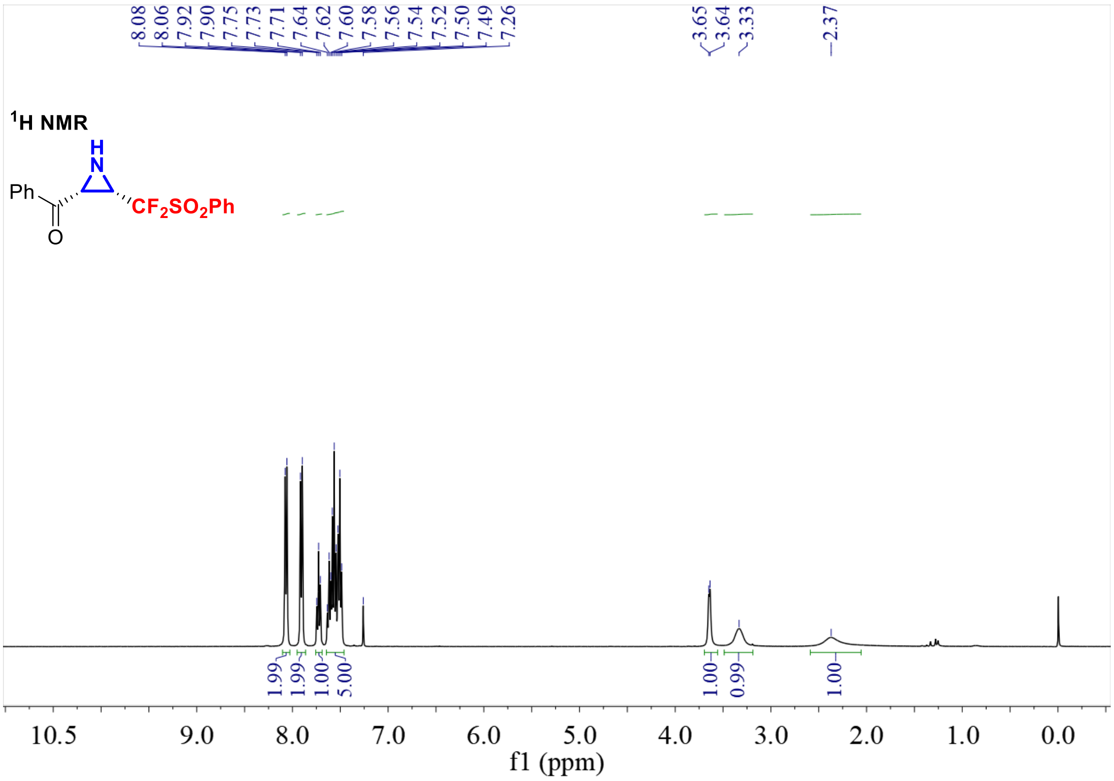
8.08
8.06
7.92
7.90
7.75
7.73
7.71
7.64
7.62
7.60
7.58
7.56
7.54
7.52
7.50
7.49
7.26
3.65
3.64
3.33
2.37
1H NMR
Ph
H
N
CF2SO2Ph
O
1.99
1.99
1.00
5.00
1.00
0.99
1.00
10.5
9.0
8.0
7.0
6.0
5.0
4.0
3.0
2.0
1.0
0.0
f1 (ppm)

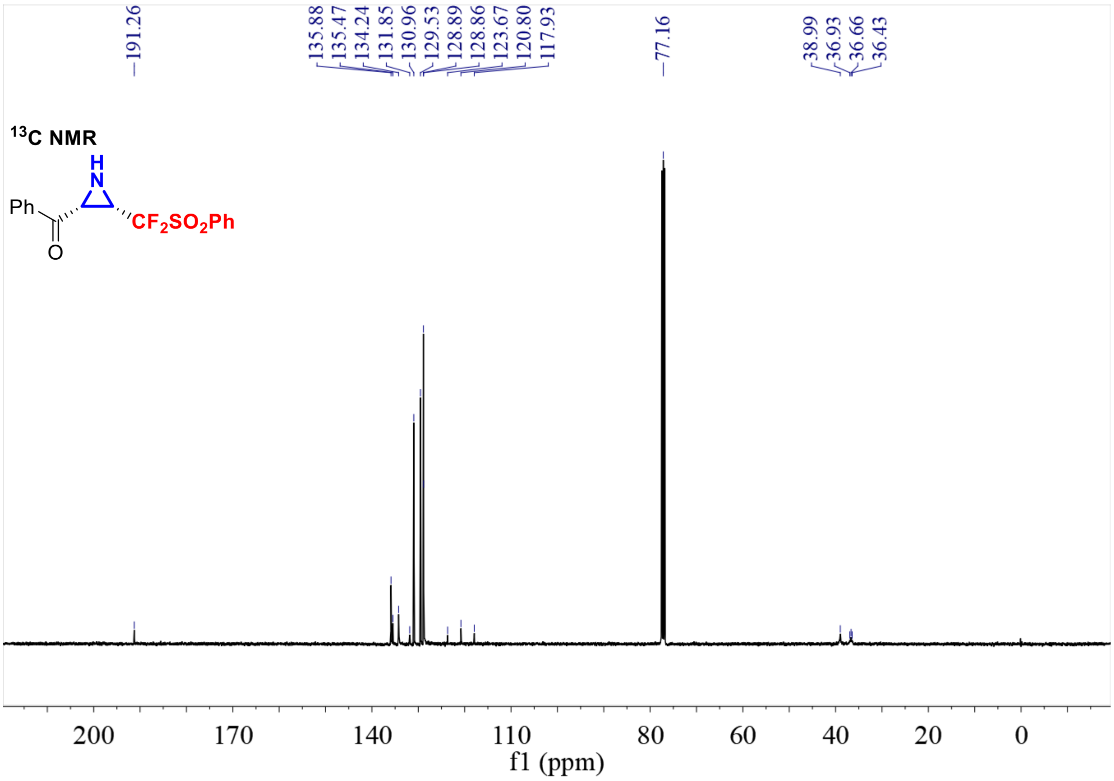
191.26
135.88
135.47
134.24
131.85
130.96
129.53
128.89
128.86
123.67
120.80
117.93
77.16
38.99
36.93
36.66
36.43
13C NMR
Ph
H
N
CF2SO2Ph
O
200
170
140
110
80
60
40
20
0
f1 (ppm)

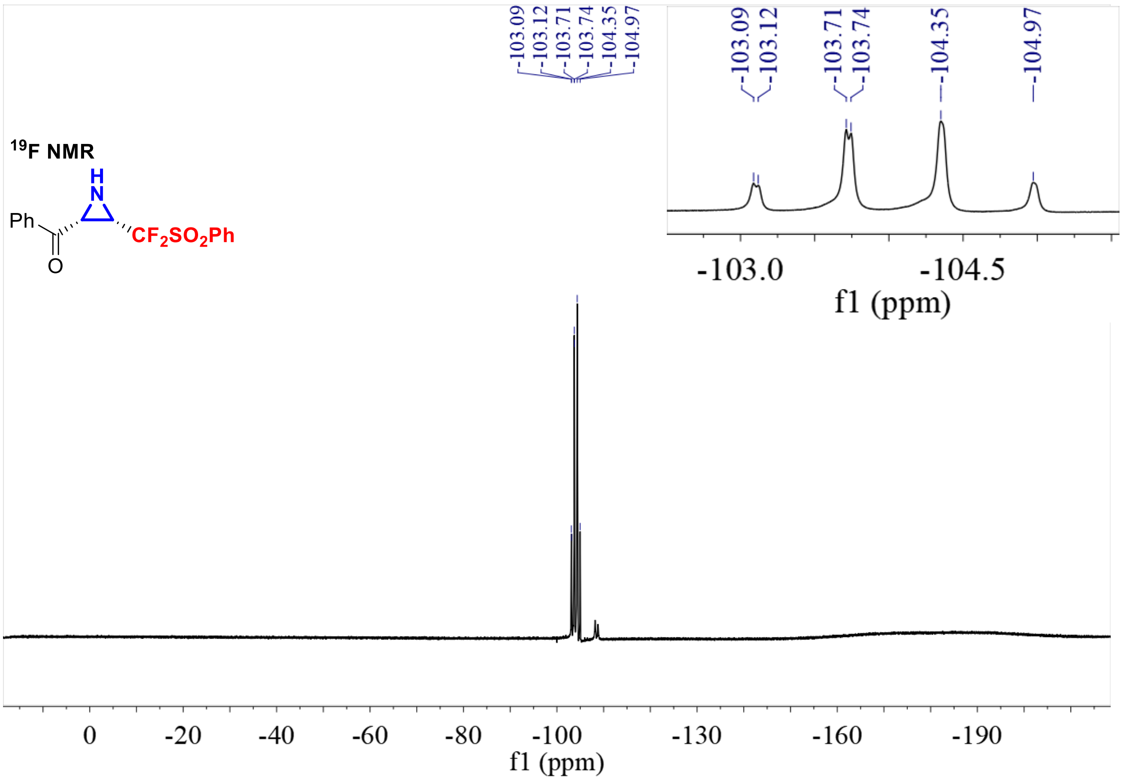

19F NMR
-103.09
-103.12
-103.71
-103.74
-104.35
-104.97
f1 (ppm)

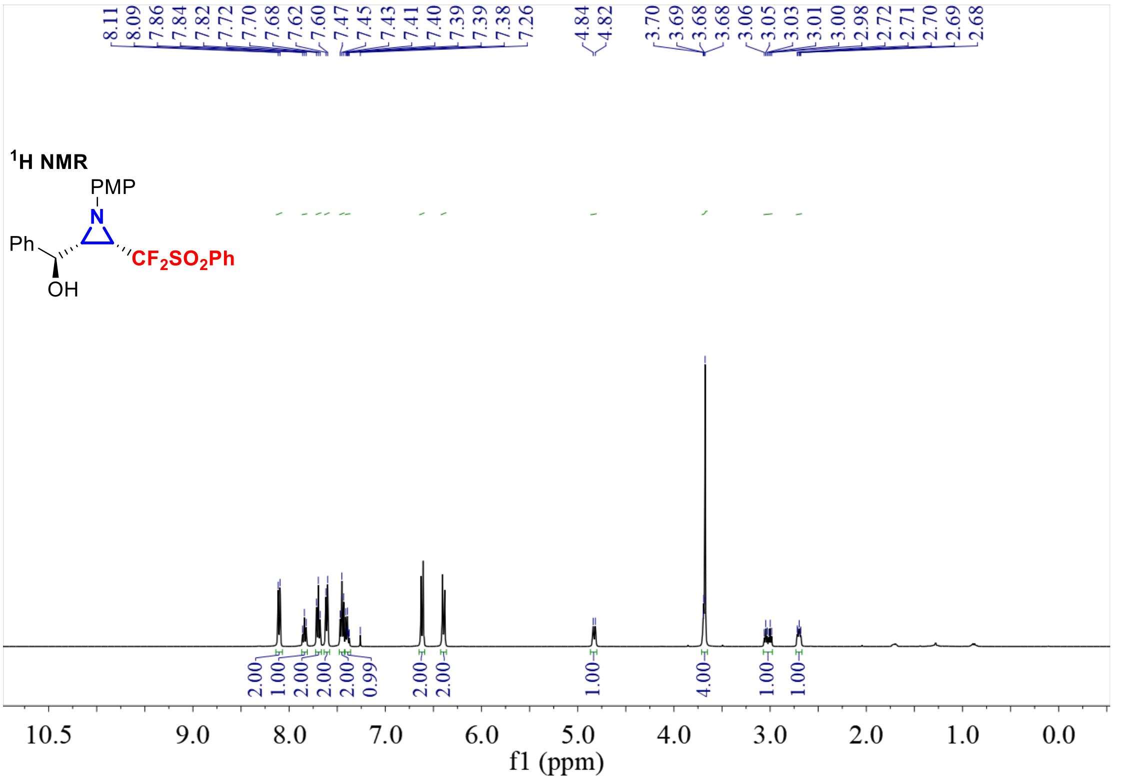

1H NMR
PMP
Ph
OH
CF2SO2Ph
f1 (ppm)

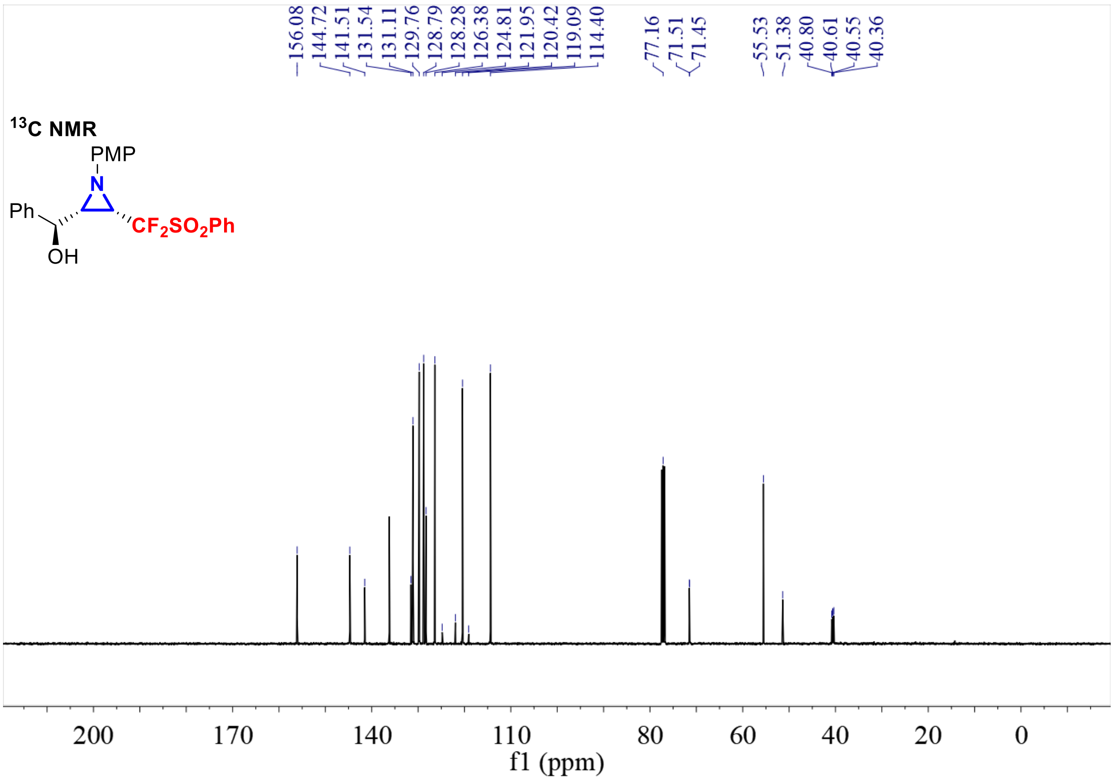

156.08
144.72
141.51
131.54
131.11
129.76
128.79
128.28
126.38
124.81
121.95
120.42
119.09
114.40
77.16
71.51
71.45
55.53
51.38
40.80
40.61
40.55
40.36
^{13}C NMR
PMP
N
Ph
OH
CF_2SO_2Ph
200
170
140
110
80
60
40
20
0
f1 (ppm)

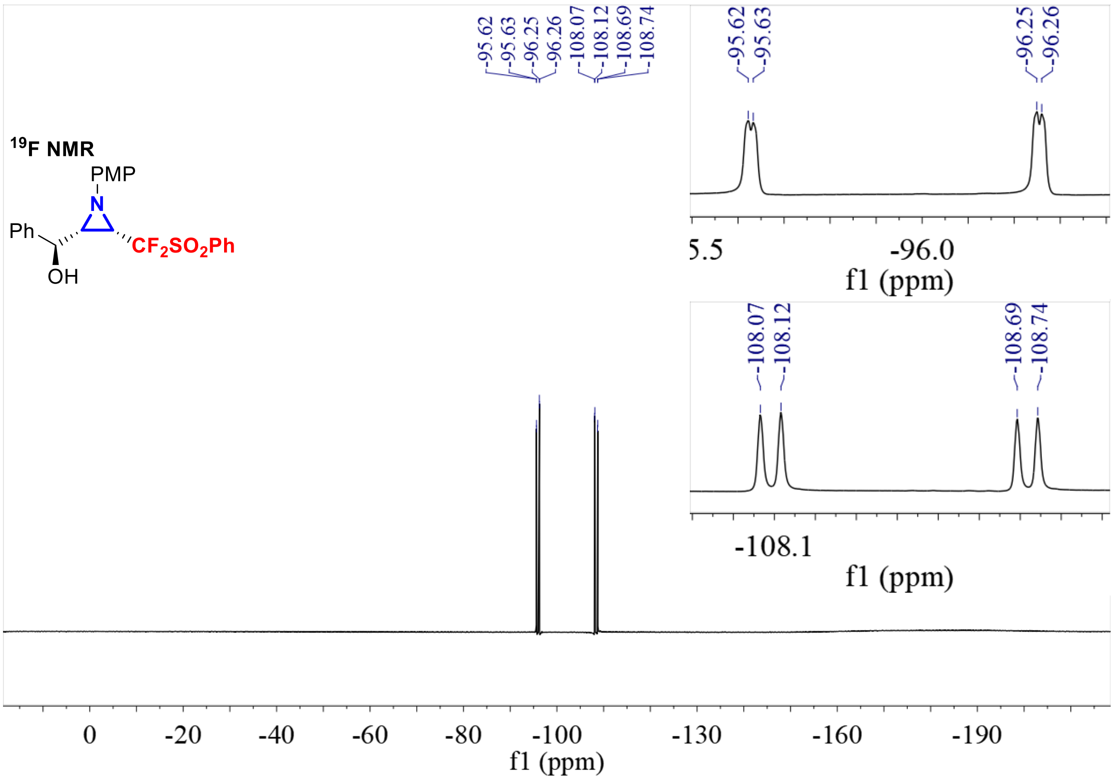

-95.62
-95.63
-96.25
-96.26
-108.07
-108.12
-108.69
-108.74
^{19}F NMR
PMP
N
Ph
OH
CF_2SO_2Ph
5.5
-96.0
f1 (ppm)
-108.1
f1 (ppm)
0
-20
-40
-60
-80
-100
-130
-160
-190
f1 (ppm)

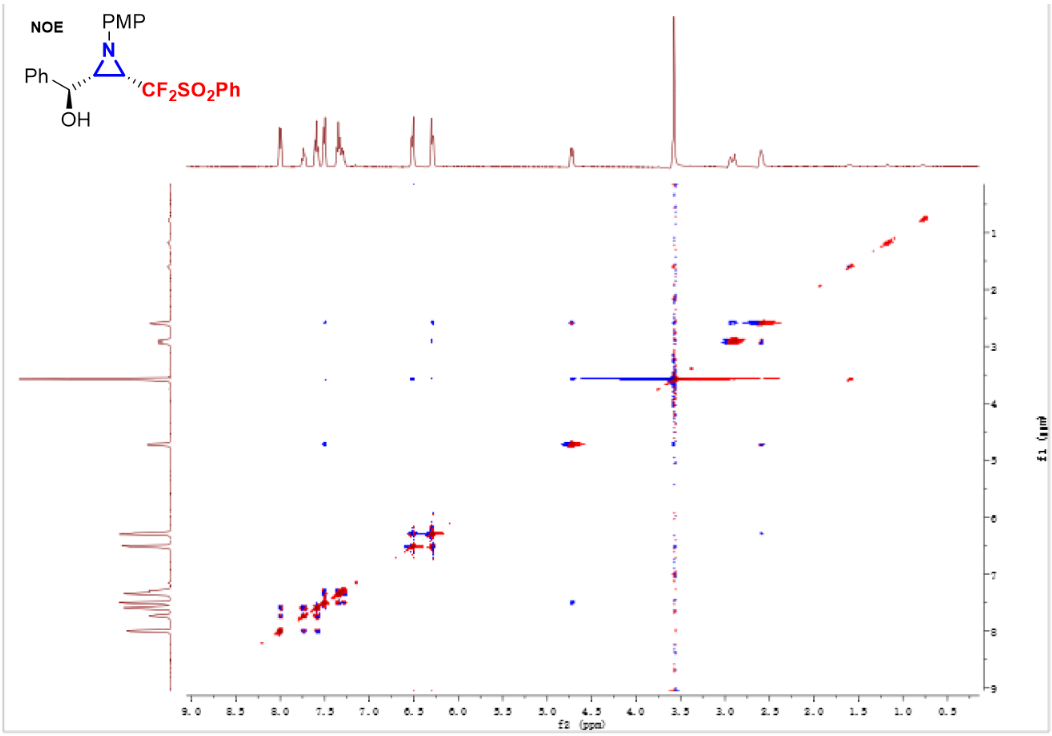
NOE
PMP
N
Ph
OH
CF2SO2Ph
f2 (ppm)
f1 (ppm)

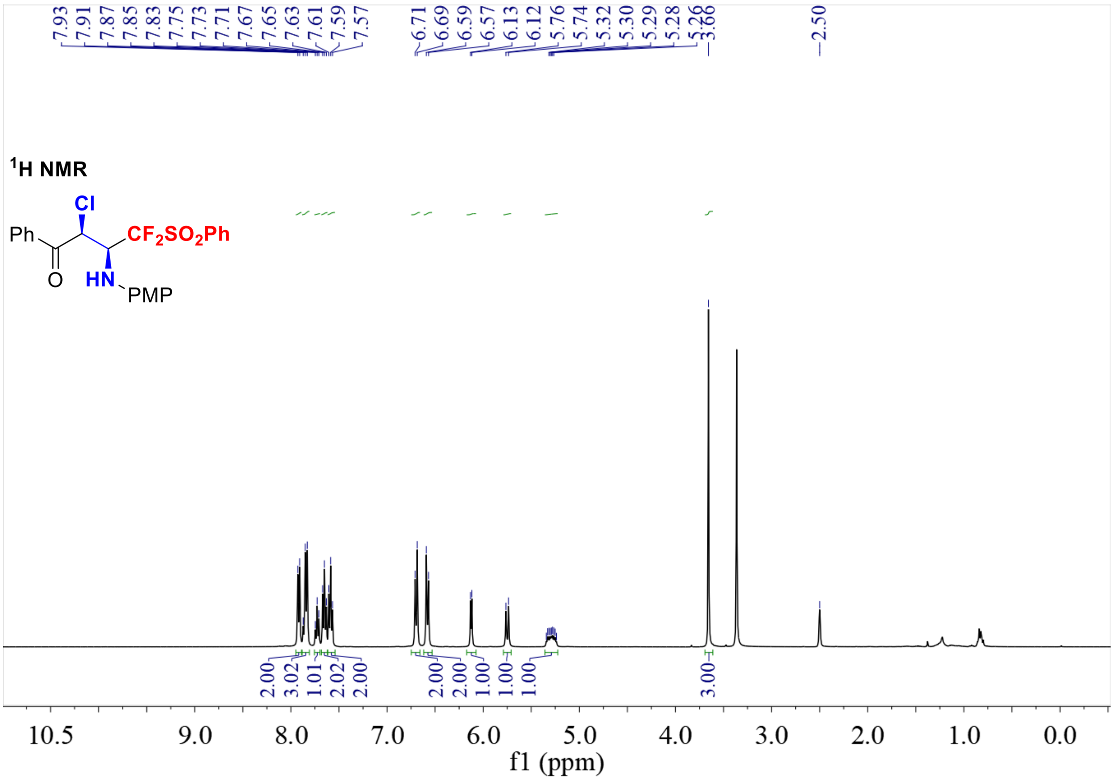
1H NMR
Cl
Ph
CF2SO2Ph
O
HN
PMP
7.93
7.91
7.87
7.85
7.83
7.75
7.73
7.71
7.67
7.65
7.63
7.61
7.59
7.57
6.71
6.69
6.59
6.57
6.13
6.12
5.76
5.74
5.32
5.30
5.29
5.28
5.26
3.66
2.50
2.00
3.02
1.01
2.02
2.00
2.00
2.00
1.00
1.00
1.00
3.00
f1 (ppm)

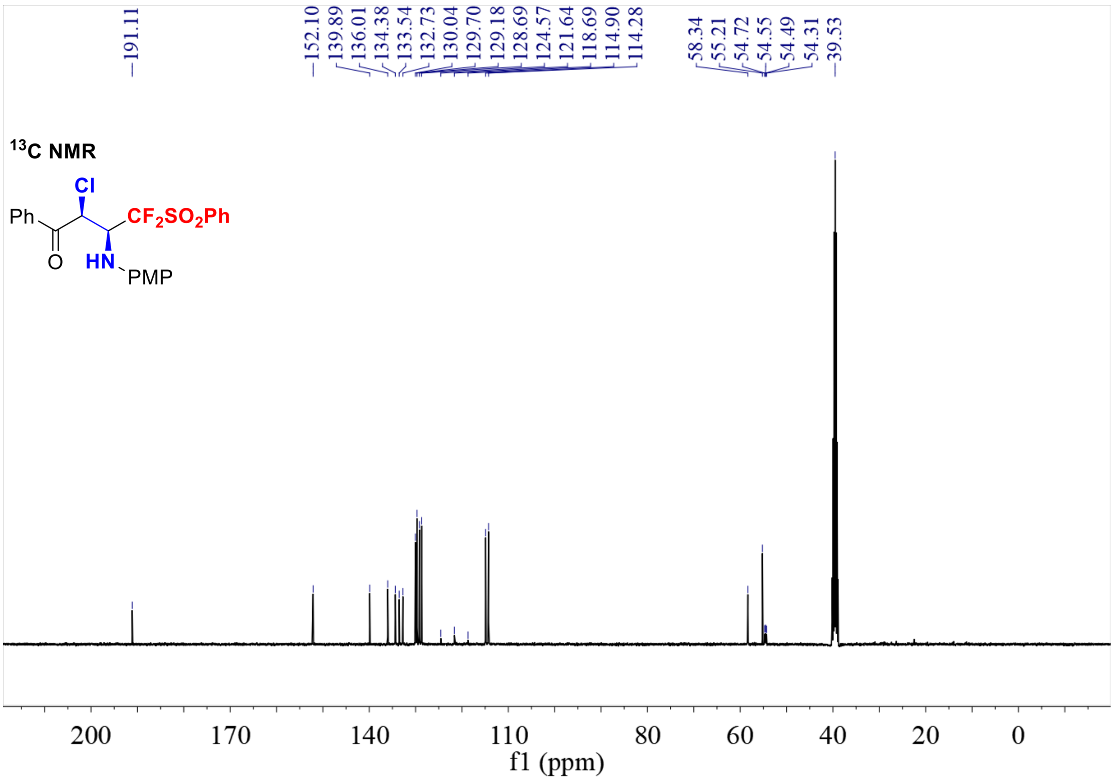

191.11
152.10
139.89
136.01
134.38
133.54
132.73
130.04
129.70
129.18
128.69
124.57
121.64
118.69
114.90
114.28
58.34
55.21
54.72
54.55
54.49
54.31
39.53
13C NMR
Cl
Ph
CF2SO2Ph
O
HN
PMP
200
170
140
110
80
60
40
20
0
f1 (ppm)

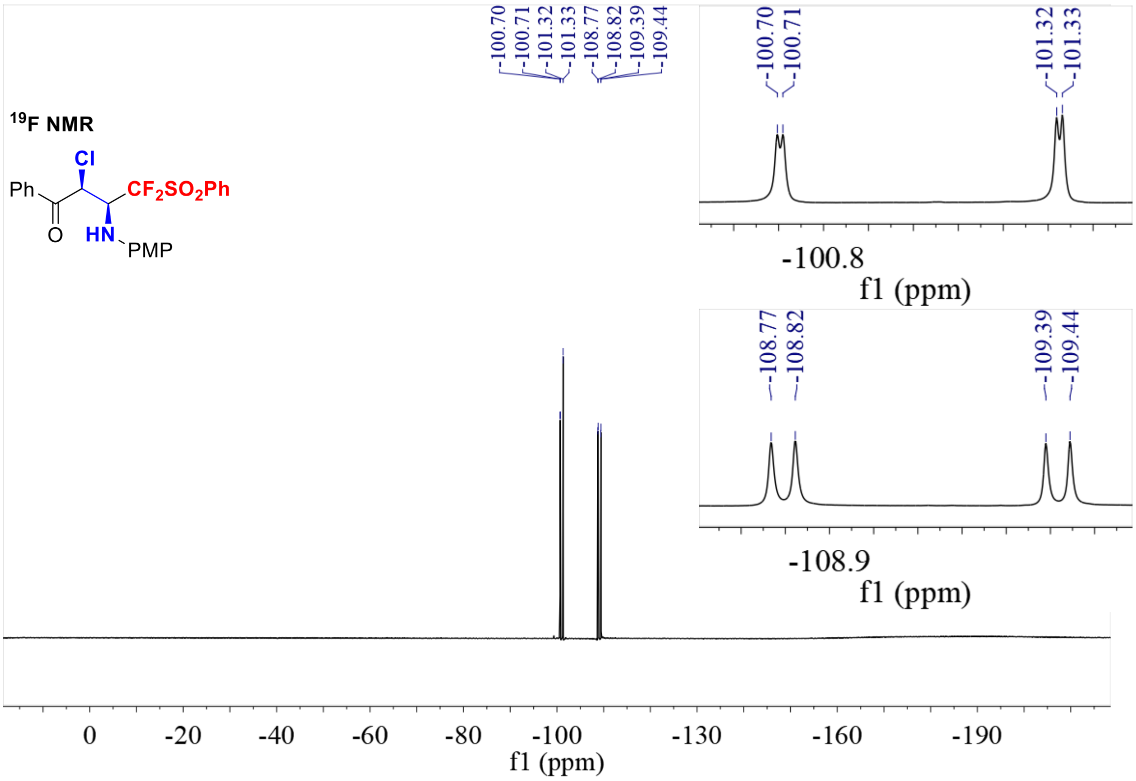

-100.70
-100.71
-101.32
-101.33
-108.77
-108.82
-109.39
-109.44
19F NMR
Cl
Ph
CF2SO2Ph
O
HN
PMP
-100.8
f1 (ppm)
-108.9
f1 (ppm)
0
-20
-40
-60
-80
-100
-130
-160
-190
f1 (ppm)

5 X-ray crystallographic data

5.1 The X-ray crystallographic structures for compound **4a**. Crystal data have been deposited to CCDC, number 1983642.

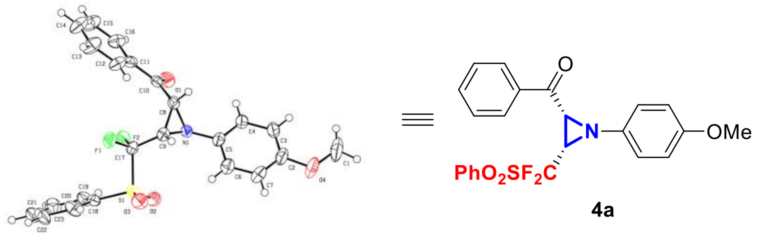

4a

Table 1 Crystal data and structure refinement for Compound 4a

Identification code	22019902TXF_0m
Empirical formula	$C_{23}H_{19}F_2NO_4S$
Formula weight	443.45
Temperature/K	173.0
Crystal system	monoclinic
Space group	$P2_1$
a/Å	8.9940 (4)
b/Å	10.3367 (5)
c/Å	11.4489 (5)
α/°	90
β/°	91.170 (2)
γ/°	90
Volume/Å^3	1064.16 (8)
Z	2
ρcalcg/cm^3	1.384
μ/mm^{-1}	1.773
F (000)	460.0
Crystal size/mm^3	0.19 × 0.15 × 0.12
Radiatio	CuKα (λ = 1.54184)
2Θ range for data collection/°	12.392 to 133.332
Index ranges	10 ≤ h ≤ 10, -12 ≤ k ≤ 11, -13 ≤ l ≤ 13
Reflections collected	8172
Independent reflections	3473 [R_{int} = 0.0278, R_{sigma} = 0.0417]
Data/restraints/parameters	3473/1/281
Goodness-of-fit on F^2	1.117
Final R indexes [I>=2σ (I)]	R_1 = 0.0302, wR_2 = 0.0771
Final R indexes [all data]	R_1 = 0.0304, wR_2 = 0.0772
Largest diff. peak/hole / e Å^{-3}	0.24/-0.29
Flack parameter	0.064 (8)

5.2 The X-ray crystallographic structures for compound **5c**. Crystal data have been deposited to CCDC, number 1983643.

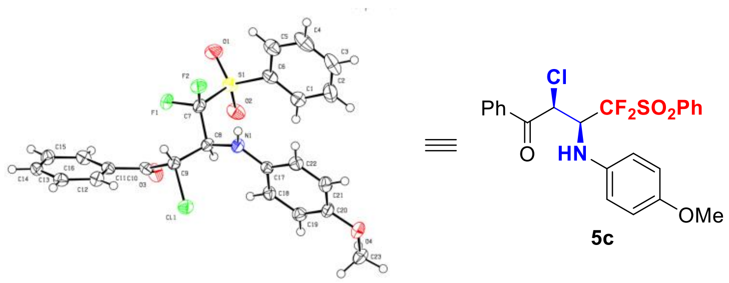

Table 1 Crystal data and structure refinement for Compound 5c

Identification code	DS
Empirical formula	$C_{23}H_{20}ClF_2NO_4S$
Formula weight	479.91
Temperature/K	170.0
Crystal system	monoclinic
Space group	$P2_1$
a/Å	5.5826 (8)
b/Å	16.132 (2)
c/Å	11.8958 (13)
α/°	90
β/°	90.12 (5)
γ/°	90
Volume/$Å^3$	1071.3 (2)
Z	2
ρcalcg/cm^3	1.488
μ/mm^{-1}	0.325
F (000)	496.0
Crystal size/mm^3	0.19 × 0.12 × 0.08
Radiation	MoKα (λ = 0.71073)
2Θ range for data collection/°	5.05 to 53.128
Index ranges	$-6 \leq h \leq 7$, $-20 \leq k \leq 19$, $-14 \leq l \leq 14$
Reflections collected	12417
Independent reflections	4273 [R_{int} = 0.0518, R_{sigma} = 0.0626]
Data/restraints/parameters	4273/1/290
Goodness-of-fit on F^2	1.035
Final R indexes [I>=2σ (I)]	R_1 = 0.0434, wR_2 = 0.0836
Final R indexes [all data]	R_1 = 0.0685, wR_2 = 0.0982
Largest diff. peak/hole / e $Å^{-3}$	0.25/-0.32
Flack parameter	-0.07 (5)
